# Cross Determination of Exciton Coherence Length in J-Aggregates

**DOI:** 10.1021/acs.jpclett.2c02213

**Published:** 2022-10-25

**Authors:** A. Jumbo-Nogales, V. Krivenkov, K. Rusakov, A. S. Urban, M. Grzelczak, Y. P. Rakovich

**Affiliations:** † Centro de Física de Materiales (MPC, CSIC-UPV/EHU), San Sebastián, 20018, Spain; ‡ Polymers and Materials: Physics, Chemistry and Technology, Chemistry Faculty, University of the Basque Country (UPV/EHU), San Sebastián, 20018, Spain; ¶ Faculty of Construction and Environmental Engineering, Warsaw University of Life Sciences, 02-776Warsaw, Poland; § Nanospectroscopy Group, Nano-Institute Munich, Department of Physics, Ludwig-Maximilians-Universität München (LMU), Munich80539, Germany; ∥ Donostia International Physics Center (DIPC), San Sebastián, 20018, Spain; ⊥ Ikerbasque, Basque Foundation for Science, Bilbao, 48013, Spain

## Abstract

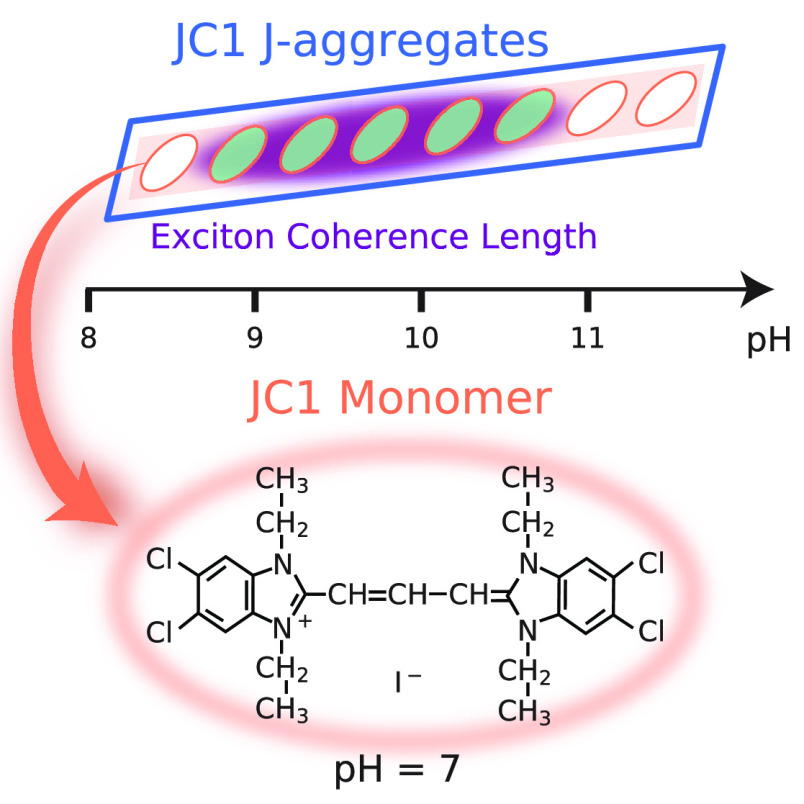

The coherence length of the Frenkel excitons (*N*_*coh*_) is one of the most critical parameters governing many key features of supramolecular J-aggregates. Determining experimentally the value of *N*_*coh*_ is a nontrivial task since it is sensitive to the technique/method applied, causing discrepancies in the literature data even for the same chemical compound and aggregation conditions. By using a combination of different experimental techniques including UV–vis–NIR, fluorescence emission, time-resolved photoluminescence, and transient absorption spectroscopies, we determined *N*_*coh*_ values for J-aggregates of a cyanine dye. We found that the absorption spectroscopy alone - a widely used technique- fails in determining right value for *N*_*coh*_. The correct approach is based on the modification of photoluminescence lifetime and nonlinear response upon aggregation and careful analysis of the Stokes shift and electron–phonon coupling strength. This approach revealed that *N*_*coh*_ of JC-1 J-aggregates ranges from 3 to 6.

J-aggregates are self-assembled supramolecular structures constituting organic molecules, organized into quasi-1D chains.^1^ Strong in-line dipole–dipole coupling in these systems leads to the generation of the Frenkel exciton, and coherence of the exciton wave function across several interconnected monomer units^2^ rendering a narrow absorption band (red-shifted with respect to monomer)—the so-called J-band.^3,4^ A quantitative description of long-range exciton migration^5^ involves the determination of coherence length *N*_*coh*_ (or the exciton coherence length). It tells about the number of molecules of an aggregate over which the exciton wave function is coherent, the dipole momentum, oscillator strength, and the radiative lifetime of the exciton transition. The coherence length also has some implications for the nature of long-distance exciton migration and transport - whether coherent or incoherent.^5^ J-aggregates gain value when combined with other systems. It has been shown that J-aggregates can reach a strong coupling regime when combined with optical microcavities or plasmonic nanoantennas.^6^ Their selectivity for biomacromolecules enables the resolving of the structural diversity in complex biological environments.^7^ Also, the exciton coherence is central to charge separation, energy transfer, and singlet fission in the molecular aggregates.^8^ Therefore, the quantification of *N*_*coh*_ is of crucial importance for further advancement in the development of J-aggregates-based hybrid systems. However, such quantification is challenging as witnessed by the discrepancies in reported values even for J-aggregates of the same compound (Table 1 in the Supporting Information). This spread may be due to the different experimental conditions during sample preparation as well as the issues and errors related to different analysis and measurement techniques. There are several methods to estimate *N*_*coh*_, depending on the analyzed data: absorbance,^9^ transient absorption,^10^ and photoluminescence time-resolved^11^ spectroscopies. And it is less common to derive *N*_*coh*_ from measurements of the Stokes shift between the maxima of the absorption and photoluminescence bands and scaling of the Huang–Rhys (HR) parameter.^12^ This parameter was derived to characterize the electron–phonon coupling strength in a crystal^13^ but it also can be applied in the description of the interaction of the molecular vibrations with electronic excitations.^14^

In this paper, we quantify the coherence length of cyanine dye as an exemplar model J-aggregates system by using four different methods. The first one is based on the estimation of the exciton coherence determining the line width length of the J-band with respect to the monomer band.^9^ The second method requires the measurement of the absorbance and emission spectra, and in this way, the *N*_*coh*_ was obtained by employing the evaluation of the Huang–Rhys factor^12^ from the difference in position of the centers of the J-band absorbance and emission small Stokes shift.^14^ The photoluminescence lifetime was also analyzed, and in this case, *N*_*coh*_ was estimated from the ratio between the monomer and J-aggregates emission rates.^12,15^ The last applied method is based on pump–probe (transient absorption) measurements. Here the difference between the one-exciton bleaching peak and two-exciton induced absorption^16,17^ allowed us to find a value for the exciton wave function spread in the J-aggregates. In each described case, we monitored the evolution of the J-band and *N*_*coh*_ over a wide range of pH following dynamics process of aggregation and obtained a similar *N*_*coh*_ value using the last three methods, while the first method gave a somewhat bigger value. With the knowledge of *N*_*coh*_, such essential parameters as the total number of generated excitons, the value of the dipole moment, and the oscillator strength on the exciton transition were obtained. Actually none of these parameters have ever been reported for J-aggregates of the cyanine dye used in our work (JC1), despite its wide use in research on strong light–matter interaction and biological applications.^12,18^

The J-aggregates were obtained after dissolving 20 μL of JC1 monomer (250 μM in ethanol solution) in 2 mL of water in a plastic receptacle. After that 20 μL of NaOH (0.1 M aqueous solution) was added to increase the pH. Once the solution reaches a pH value over 8, it acquires a light pink color due to the presence of the J-aggregates.

*Characterization.* The absorption spectra of the samples were taken using Cary 3500 UV–vis spectrophotometer, the emission spectra were measured using Cary Eclipse Fluorescence Spectrophotometer (Agilent Technologies). The PL decays were acquired using a time-resolved confocal fluorescence microscope MicroTime 200 and a laser excitation of 485 nm. And the PL response of monomer and J-aggregates were separated using two filters of 510 and 593 nm, respectively. The lifetimes of the JC1 monomer and J-aggregates were estimated by fitting the photoluminescence (PL) decay curves with SymPhoTime 32 software. The analysis of the J-aggregates with pump–probe was done with a custom-built transient absorption spectrometer from Newport Inc. The Ti:Sa amplifier was operating at 1 kHz (Libra from Coherent Inc.) and it acted as a light source. An OPA system (Opera Solo from Coherent Inc.) generated the excitation beam with a spot diameter of 350 μm, a pulse duration of 100 fs, and typical excitation powers of 13–55 μJ/cm^2^. All these measures were performed under ambient conditions.

*Estimation Methods.* To determine the value of the coherence length of JC1 aggregates, we have implemented four different approaches, using data from several spectroscopic methods: UV–vis absorbance, transient absorption, emission spectra, and PL lifetimes.

The JC1 monomer molecule absorbance spectrum shows a maximum at 2.43 eV, and a shoulder at 2.2 eV. Its emission spectrum is almost a mirror image of the absorbance, however, with a maximum red-shifted by 0.11 eV (Figure 1 a). These spectra change when the monomer molecules aggregate forming chains. Then, the shift between the absorbance and PL response is notably small and can even be considered nonexistent.

**Figure 1:**
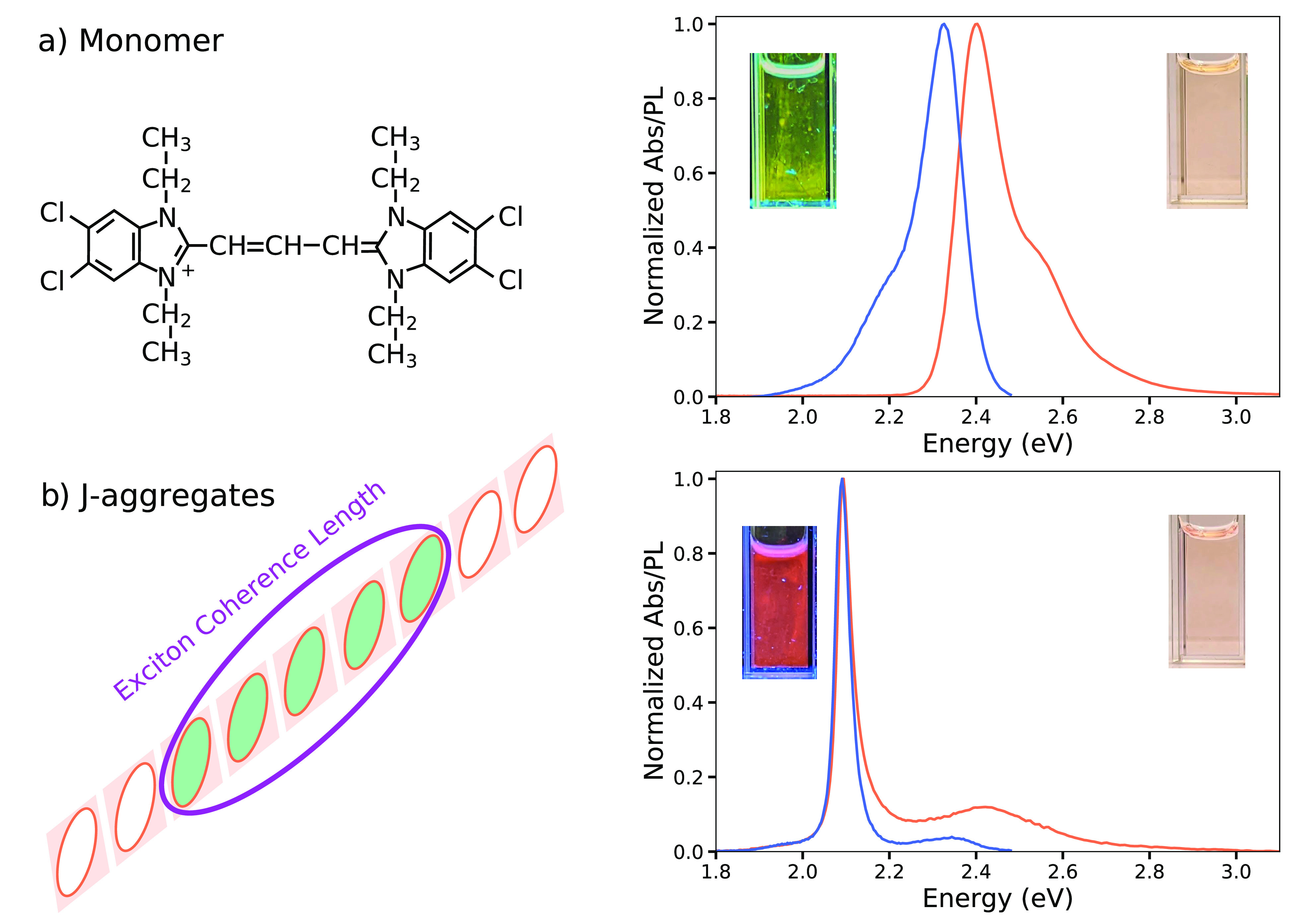
Monomer and J-aggregate absorbance and photoluminescence characterization. (a) JC1 molecular structure (left). Absorbance spectrum of the JC1 monomer molecule (orange) with maximum at 2.4 eV and a shoulder at 2.48 eV; emission spectrum (blue). Inset: digital images of monomer’s solution under UV illumination (left) and ambient light (right). (b) Schematic representation of the J-aggregate (monomer chains)and exciton coherence length (left). Absorbance (orange) and emission (blue) spectra of the J-aggregates, showing a narrow peak with a maximum near to 2.08 eV.

J-aggregates appear once NaOH is added to JC1 in an aqueous solution due to an increase in the pH. The molecular chain formation can be perceived by the naked eye because of the light pink color acquired by the initially transparent solution. This pH influence in the aggregation of the JC1 molecules was analyzed through four experiments (see the Supporting Information, Figures S1, S2, S3, and S4).

The evolution of the J-aggregates’ absorbance and emission spectra as a function of solution pH is presented in Figure 2. In both spectra, at low pH, only the signature of the monomer is present. The signal from the J-band rapidly grows with increasing pH. But, while the absorbance saturates for pH > 11, the PL intensity reaches a maximum at pH = 10.5 and then rapidly decreases.

**Figure 2:**
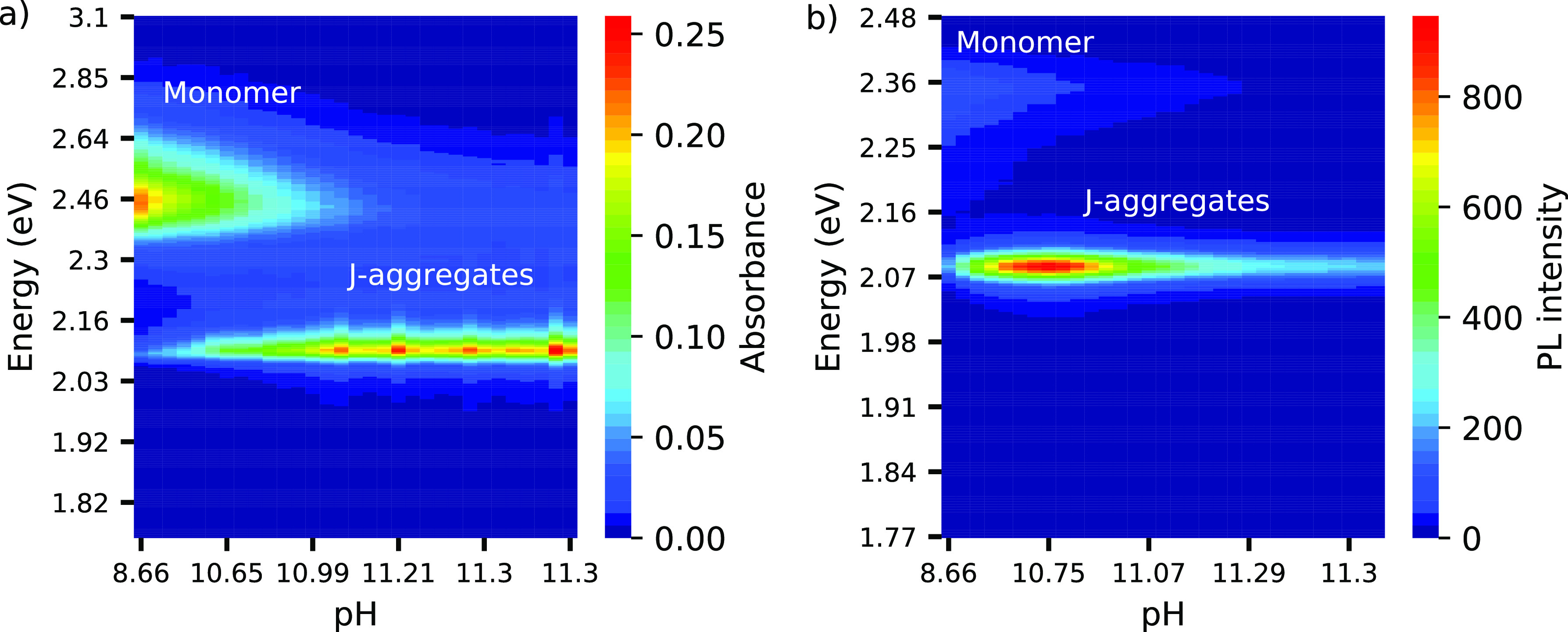
pH-dependent formation of J-aggregates. The pH was increased from 8.6 to 11.3 in sequential addition of NaOH (440 μL in 21 steps). (a) Absorbance spectra. With increasing NaOH, the J-band progressively increases accompanied by decrease of monomer band. (b) Emission spectra. The emission from monomer decreases with pH while PL of the J-band appears immediately after the first addition of NaOH. The intensity of PL from the J-aggregate decreases for pH > 10.5.

The absorbance response of the J-aggregates during their formation with pH increment was monitored every 2 min. The J-band grows with pH value, becoming more intense and narrower than the monomer band. This behavior is due to molecular aggregation and the lower concentration of single molecules. It was established that the line width modification of these bands is generally related to *N*_*coh*_.^19^ However, this approach for estimating *N*_*coh*_ was not seen as too accurate and precise because the bandwidth of the monomers forming J-aggregates may differ from that of monomers.^20,21^ Alternatively, Bakalis and Knoester take into account that the J-aggregate absorption bandwidth determines the energy separation between the two lowest states on one localization segment, and relate it to *N*_*coh*_. They used numerical simulations to compare the results of both methods, concluding that in the best approach *N*_*coh*_ can be estimated by eq 1.
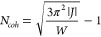
1

This relation is established after analyzing linear molecular aggregates built by N molecules of two levels, considering the static disorder, and in a regime where the coherence length is smaller with respect to the aggregate size. In our analysis, the width *W* of the JC1 J-aggregates was obtained by fitting each absorbance spectra to a Voigt curve (Figure 3a). This profile is a convolution of a Gaussian and a Lorentz distribution and was chosen because of the asymmetrical shape of the J-aggregate spectra. The parameter *J* represents the dipole–dipole interaction strength in a molecular chain; it comes from the difference in maximum absorption wavelengths of J-band and monomer,^19^ and eq 2 was applied to estimate its value.
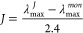
2

**Figure 3:**
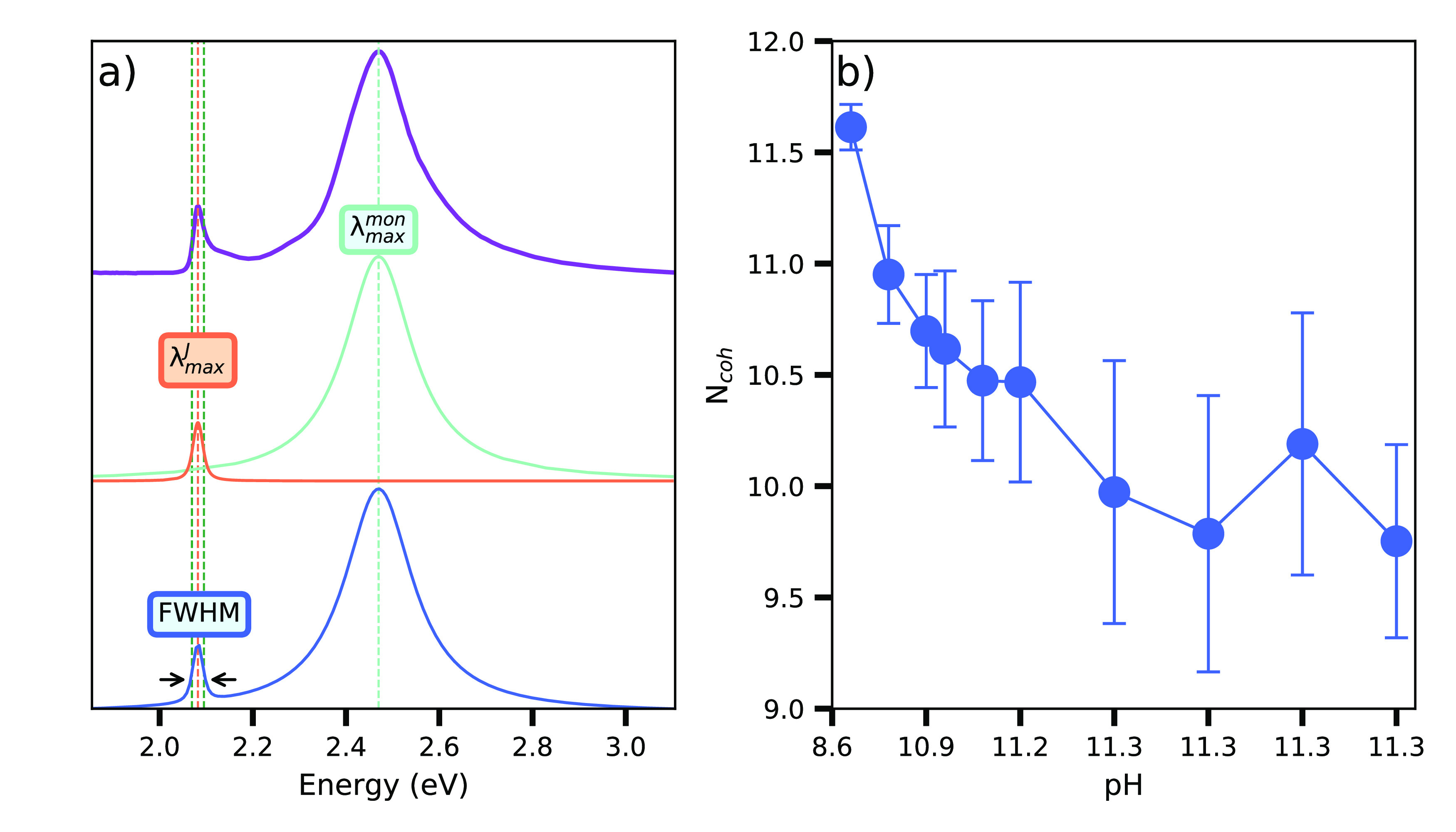
(a) Experimental absorbance spectrum of JC1 in water taken at pH = 8.6 (violet). Voigt fits of the monomer absorbance (aquamarine), the J-band absorbance (orange), and the entire spectrum (blue). (b) pH-dependent *N*_*coh*_ estimated using eq 1 and the absorbance spectra. Each point corresponds to an exciton coherence value related to the pH of solution.

This equation describes the molecular interaction in the chain. The value of 2.4 corresponds to the shift originated by the application of open boundary conditions and the inclusion of long-range interactions in the bottom exciton band.^22,23^ The justification for eq 1 arises from the increase in *N_coh_* with the suppression of exciton-vibrational coupling and hence with a narrowing of the absorption band. However, the use of eq 1 must be taken with caution because the correct measurement of the J value from the absorption shift of the J-band (eq 2) requires accounting for the ground state level shift of the molecules.^20^

Equations 2 and 1 allow us to obtain the value of *N*_*coh*_ using the analysis of the J-aggregates’ absorbance spectrum. The average coherence length obtained using this method is 10.5. The evolution of this parameter is presented in Figure 3 b); in general, *N*_*coh*_ decreases with pH, meaning that the exciton is coherent over more molecules for lower pH values. The errors associated with *N*_*coh*_ in Figure 3b) stem from the difference between the fitted spectra and the measured ones and reflect the asymmetric shapes of the monomer and J-band responses.

The J-aggregates’ PL can provide information about the exciton coherence using the Stokes shift and radiative lifetime estimation. The Stokes shift is usually calculated as the difference between absorbance and emission maxima and is related to the dimensionless Huang–Rhys (HR) parameter, which is a measure of the electron–phonon or electron–vibrational coupling strength.^24^ Because of the ambiguity of the relationship between the Stokes shift and the HR parameter used in different papers, Jong et al. presented a more advanced analysis for the influence of thermal effects in the band maximum position and how to correct for possible errors due to these effects. It is preferred to take the peak center of mass (*E*_*bc*_) to solve the fitting problems derived from its asymmetric shape.^25,26^ The values of the energy barycenter (*E*_*bc*_) can be estimated using the experimental absorption and emission spectra (*W*(*E*))^14^ as presented in the following equation:
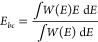
3The HR parameter (*S*) provides information about the microscopic details of the vibrational coupling. Moreover, it can be related to the equilibrium position offset between the ground state and the excited state^14^ by the following equation, where the difference between the energy barycenter of emission and absorbance spectra gives us the value of Δ*E*_*bc*_.
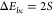
4

The coherence length can be obtained using the scaling relation^12^
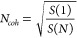
5where *S*(1) represents the monomer Huang–Rhys parameter and *S*(*N*) the J-aggregates’ HR parameter. This latter equation allows us to determine *N*_*coh*_ values from monomer and J-aggregate absorbance and emission data. The rationalization of this equation is that the shape of the spectral lines and the line width (which in turn is defined by the value of *N*) depend on the vibrational coupling strength and hence on *S*.^24^ The estimated value of Δ*E*_*bc*_ for the JC1 monomer is 0.20 eV, which is five times greater than the maximal value of the J-aggregates of Δ*E*_*bc*_ = 0.04 eV (Figure 4a).

**Figure 4:**
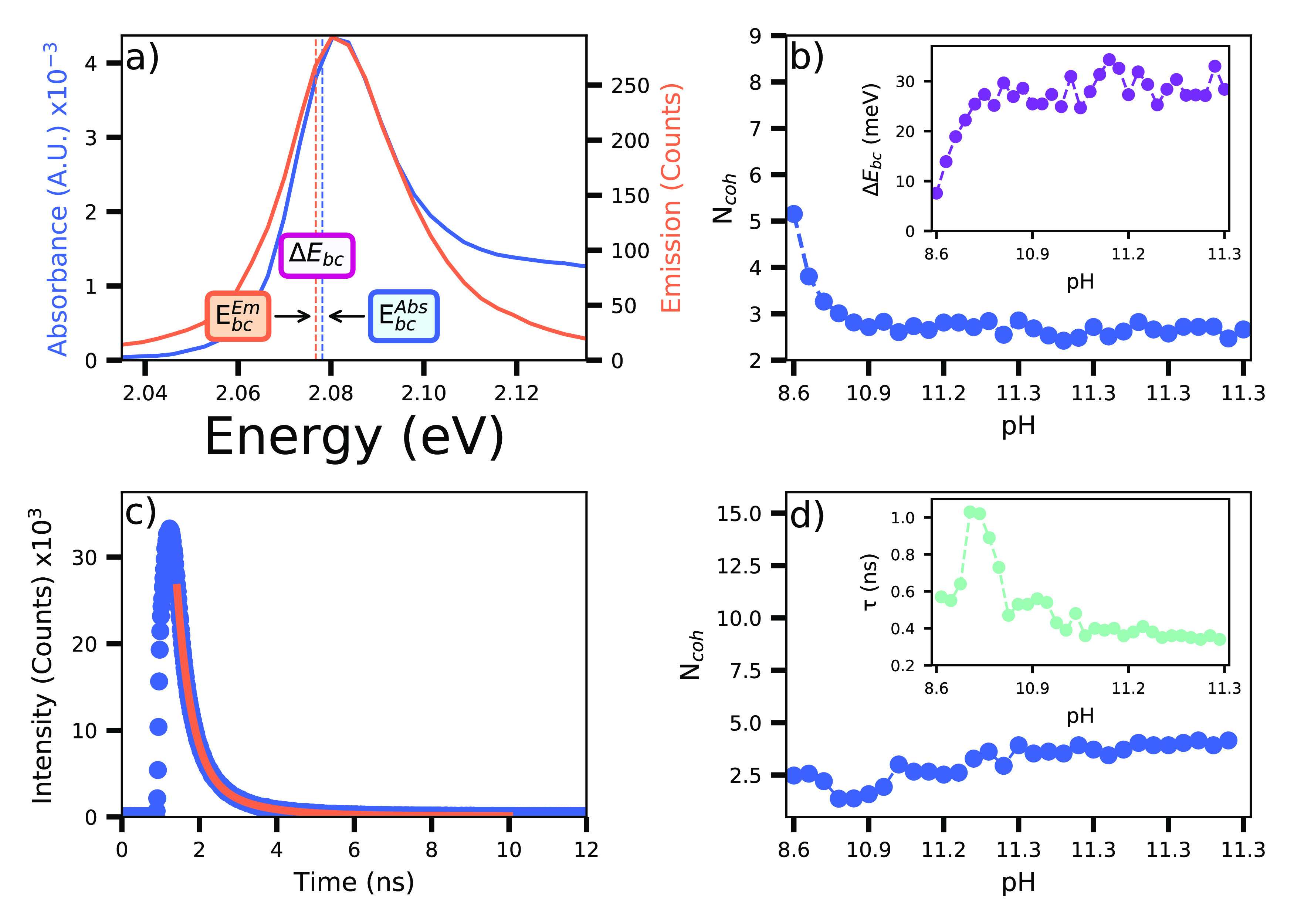
Results of analysis of J-aggregate absorption and PL to calculate the exciton coherence length: (a) Stokes shift Δ*E*_*bc*_ estimation using energy barycenters of absorbance (blue) and emission (orange) spectra. (b) Evolution of the coherence length (estimated from Huang–Rhys parameter) with pH. (c) Photoluminescence intensity decay (blue circles) and the result of the double exponential fitting (orange line) of JC1 solution with pH = 8.6. (d) Photoluminescence lifetimes (τ) of J-aggregates (aquamarine dots) and *N*_*coh*_ calculated from radiative decay rates of monomer and J-aggregates in function of pH.

The value of the coherence length obtained from *S* is 3 on average (Figure 4b). The value of *N*_*coh*_ decreases with J-aggregate concentration. The absorbance spectra reveal the maximal J-aggregate concentration at pH 11.2 (see Figure 2a). The first value of *N*_*coh*_ is the highest (5.2) and the final one (2.6) is reached at the highest pH (11.3). Conversely, the first Δ*E*_*bc*_ value is the lowest (7 meV), increasing up to almost 35 meV. Evolutions of these quantities are shown in Figure 4b). The dispersed *N*_*coh*_ values found in this analysis could be due to the effect of pH changes induced by the stepwise addition of NaOH to the J-aggregate absorbance and emission spectra. However, we must take into account that in the model used to link the Huang–Rhys parameter with the coherence length value, we consider the line shapes of absorbance and photoluminescence spectra to be mirror-image symmetric. In general, this is not completely correct for J-aggregates, as these spectral observables result from different excitonic properties. While the absorbance response is related to the exciton bandwidth, the PL is associated with the exciton coherence length *N*_*coh*_.^27^

The radiative and nonradiative rates determine the ratio of emitted and absorbed photons, they are constant and independent. In this analysis, we use the relation between the coherence length value and the radiative rate due to the dipole moment enhancement in the molecular chains.^28^ The quantum yield (Φ) for JC1 monomer and J-aggregates can be estimated comparing their photoluminescence with widely known dyes luminescence. For monomer quantum yield we used Rhodamine 6G (Φ = 0.95), and we obtained Φ = 0.05. In the case of J-aggregates, the photoluminescence analysis of Rhodamine 6G and quantum dots (Φ = 0.4) allowed us to obtain Φ = 0.3. The measured average lifetime (τ) of J-aggregates is 0.5 and 0.18 ns for monomeric PL.

The scaling relation of the radiative lifetime^12^ was applied to obtain the coherence length using eq 6. According to the seminal work of Kasha,^29,30^ the dipole moment of a linear chain of molecules (i.e., J-aggregates) increases as μ_*J*_ = (*N*_*coh*_)^1^/^2^μ_*J*_, where μ_*J*_ and μ_*mon*_ are the dipole moments of the aggregate and monomer, respectively. Since the radiative rate constant for the dipole-allowed transition can be expressed as *k*_*rad*_ = 4 μ^2^/3*ℏ*^3^λ^3^*c*^3^, *N*_*coh*_ can be found using radiative rate constants of the exciton and monomer based on the measurement of emission decay.
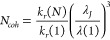
6

Equation 6 shows the relation between the coherence length, the radiative rates of J-aggregates *k*_*r*_(*N*), and the monomer *k*_*r*_(1), and the emission wavelengths of J-band λ_*J*_ and monomer λ(1). To obtain the radiative rate of monomer and J-aggregates from the lifetime value, we applied eq 7.^31^
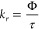
7The applicability of eq 6 can be limited by warping affecting the molecules in general. Such deformation can oppose the coherence of the exciton.^32^

The coherence length of J-aggregates, in the case of measured lifetime analysis, is *N*_*coh*_ = 3. The corresponding values are presented in Figure 4d). The results in both estimations exhibit a similar average value, and can be considered close to the ones obtained using the emission spectra (Figure 4b) from the Huang–Rhys parameter, which was expected as they are also related to J-aggregate PL.

After the estimation of *N*_*coh*_, taking into account the pH’s variation in the solution and using absorbance and PL analysis, we established that the coherence length decreases with pH value. Also, it is important to point out that the intensity of the PL response (see Figure 2) dropped after a certain pH value. Both phenomena can be related to nondiagonal and diagonal J-aggregate disorder.^33^

There is another method related to PL response and *N_coh_*. It is established that it is possible to apply the PL line strength ratio to evaluate exciton coherence length at room temperature as long as *k*_*b*_*T*, σ(disorder) ≪ ω.^34^ Although this method presents a very reliable way to obtain the exciton coherence length, the study of coherence length on temperature and extent of disorder is beyond the scope of our work, and these parameters are not among the variables we consider. We plan to do such research in the future.

The J-aggregates’ pump–probe spectra (Figure 5a) are characterized by two peaks: one corresponding to the J-band response (where ΔOD is negative) at 2.1 eV, and a second one (ΔOD is positive) in the range of 2.13–2.25 eV. The last is due to a cascaded biexcitonic transition that causes photoinduced absorption at higher energies.^35^ The negative contribution in the spectra comes from the transition of excited single exciton to two exciton states, and it produces ground state bleaching and stimulated emission. The pump–probe spectra of J-aggregates show a separation Δ (Figure 5b), which is the blueshift between the two dominant transitions (Ω_*k* = 1_^0^ corresponding to the single exciton bleaching peak and Ω_*k* = 2_^0^ the one- to two-exciton induced absorption) in the pump–probe spectrum.^16^eq 8 relates this value to the nearest-neighbor transfer interactions *J* and *N*_*coh*_.^17^
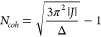
8

**Figure 5:**
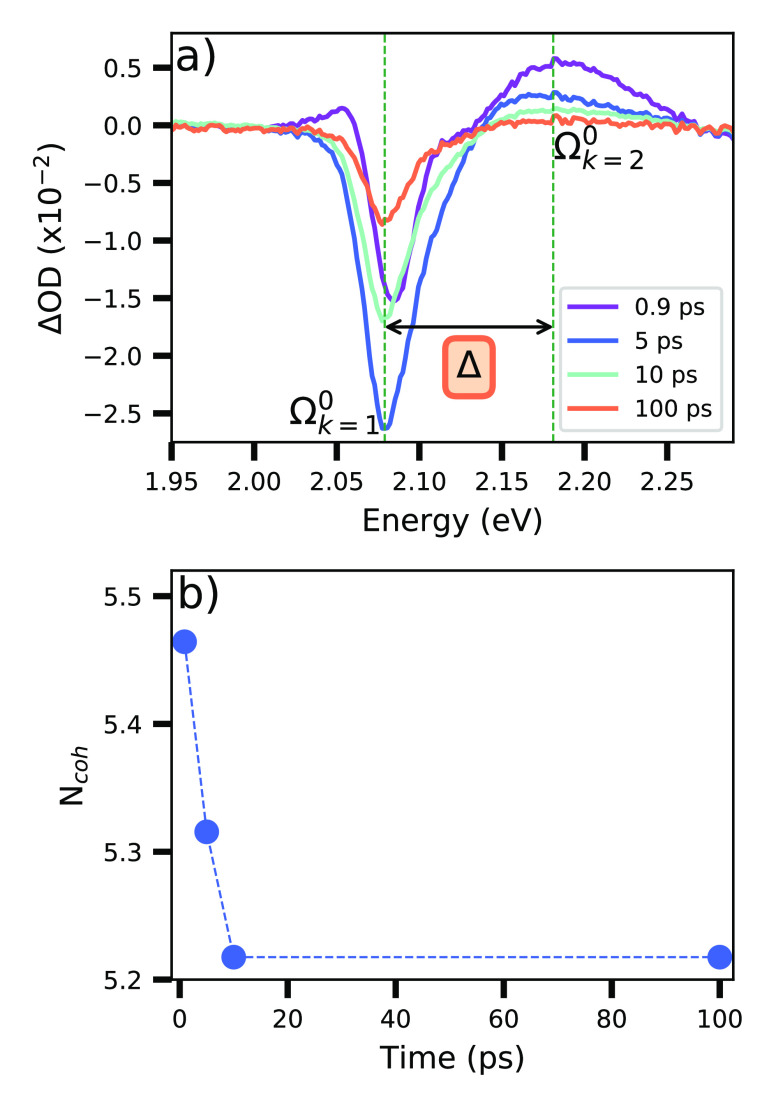
Estimation of *N*_*coh*_ from transient absorption measurements. (a) Pump–probe spectra of J-aggregates for different delay times between probe and pump laser and the spectral separation Δ*f*or the spectrum taken at 5 ps delay time. (b) *N*_*coh*_ values determined for the spectra presented in part a.

The term Δ is defined by the following relation:
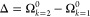
9The limits of eq 8 application are given by spectrum saturation: the pump–probe spectrum shape is dominated by homogeneous line width. The origin of this saturation is the overlap of bleaching and induced-absorption contributions due to further disorder decrease.^17^ Then, Δ is related to the saturation length.^16^

Equations 8 and 9 allowed us to estimate the coherence length of the J-aggregates (Figure 5b). The obtained value of *N*_*coh*_ on average is 5.3. It was calculated from the results of each spectrum taken at different delay times between the pump and probe laser.

After the analysis of the absorption, PL, lifetimes, and transient absorption results (summarized in Table 1), the value of *N*_*coh*_ can be established as 4 for JC1 J-aggregates. In the case of the J-band line width associated calculation, the obtained values are higher than the other estimated ones that were found using different methods. This difference can be attributed to the direct influence of the irregular band shape in the absorbance spectra. Looking at some reported values of coherence length for J-aggregates of other molecules,^10,11,32,36−60^ we found concordance in the coherence length values with those obtained for JC1 (Table S1 in the Supporting Information). The closest values to the reported in this work are the estimated for TDBC,^36^ DMDC^38^ and PIC.^37^*N*_*coh*_ = 4 was reported for all three, also a coherence length value of 6 was found for TDBC^43^ (using photoluminescence decay analysis) and for thyacarbocyanine.^47^ The pH relation with the exciton coherence length of J-aggregates can be explained by their oxidation. The J-aggregates become oxidized in acidic environments (small pH values), which leads to a drop in absorbance and fluorescence. This makes acidic conditions inherently disruptive for the coherence length. In contrast, oxidation becomes no longer possible under basic conditions (up to about pH 10), where we found that the *N*_*coh*_ value stabilizes.

**Table 1:** Summary of the Applied Methods for JC-1 J-Aggregates *N*_*coh*_ Determination: *N*_*coh*_ Value, Advantages, Shortcomings, and References

technique	*N*_*coh*_	advantages	shortcomings	ref
absorbance J-band’s line width	10.5	only requires absorbance spectrum	error from J-band line width estimation	(9)
Huang–Rhys parameter	3	avoid thermal effects influence	considers absorbance and PL line shapes mirror-image symmetric	(14, 27)
PL lifetimes	3	only PL properties analyzed: lifetimes, quantum yield and emission wavelengths	warping effects on the molecules	(12, 28−30)
transient absorption	5.3	only requires TA spectrum	spectrum saturation and sophisticated equipment is required	(16, 35)

On the other hand, it is possible to estimate the exciton concentration from the obtained *N*_*coh*_ value. We know the concentration of monomer molecules in the original solution (*n*_*m*_) is ∼1.24 × 10^18^ [monomer mol/m^–3^] and that the exciton extends over ∼4 monomer molecules. With these data, we found that there are 3.1 × 10^17^ excitons per cm^–3^ in our solution. The importance of the exciton concentration relies upon its influence on the luminescence efficiency,^61^ the mainly leveraged property of J-aggregates in applications.

Finally, to complete our analysis we have estimated the value of the exciton oscillator strength using the exciton’s absorption cross-section (σ_*J*_), both of which were never reported for JC1, using the following equation:
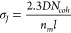
10Here *D* is the J-band absorption maximum and *l* is the light path length (1 cm). Taking *N*_*coh*_ = 4 in eq 10, we obtain σ_*J*_ = 1.05 × 10^–19^ [cm ^2^] (using the mean value of the J-band maximum). Then, we can calculate the transition dipole moment with σ_*J*_, the vacuum permittivity ϵ_0_, the Planck constant *h*, the fwhm of the J-band (using the mean value of the estimated fwhm), and the resonance wavelength λ:
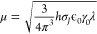
11

Using μ = 8.5 × 10^–59^[Cm] obtained from the latter equation, the mass of the electron *m*, the frequency of the J-band absorption maximum *ν*, and the electron charge *e* it is possible to estimate the oscillator strength applying eq 12:
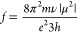
12

We found *f* = 0.04 for JC1 with *N*_*coh*_ = 4, which is a comparable value with the reported oscillator strength for PIC J-aggregates (*f*_*PIC*_ = 2.94 with *N*_*coh*_^*PIC*^ = 347) since they present an absorbance maximum at a similar wavelength.^62^ The ratio =0.008 (for JC1 and PIC) helps to illustrate the latter comparison.

The excitonic properties of JC1 J-aggregates were studied using several different spectroscopic techniques: absorption and transient absorption spectra, and PL spectra and PL lifetimes. Our work shows that a correct and reliable determination of the exciton coherence length requires a careful and comprehensive approach. This cannot be done using single-experimental analysis at fixed experimental conditions. The analysis of all the data obtained from each one of the mentioned techniques allowed us to build a better picture of the aggregation process and the excitonic behavior in the monomer chains. Finally, the calculated parameters are presented to quantify these J-aggregate properties in order to strengthen their current applications and to help develop new ones.

## Supporting Information Available

Analysis of the pH influence in the JC1 J-aggregates formation using absorbance and photoluminescence spectra and estimation of the mean lifetime of monomer and J-aggregates, results of the pH influence in the exciton coherence length applying the four explained methods to different experiments, and summary of reported J-aggregates’ exciton coherence length (PDF)
Transparent Peer Review report available (PDF)
