# Supplementary material for: Cross Determination of Exciton Coherence Length in J-Aggregates
Source: J Phys Chem Lett. 2022 Oct 25;13(43):10198–206. doi: 10.1021/acs.jpclett.2c02213 (PMC10401724; doi:10.1021/acs.jpclett.2c02213)
Supplement: Supplementary file 1 — jz2c02213_si_001.pdf [file jz2c02213_si_001.pdf]

Supporting Information
Cross Determination of Exciton Coherence Length in J-aggregates

A. Jumbo-Nogales,[†] V. Krivenkov,[†,‡] K. Rusakov,[¶] A. S. Urban,[§] M. Grzelczak,[‖] and Y. P. Rakovich*,[‖,‡,⊥]

†*Centro de Física de Materiales (MPC, CSIC-UPV/EHU), San Sebastián, 20018, Spain*

‡*Polymers and Materials: Physics, Chemistry and Technology, Chemistry Faculty, University of the Basque Country (UPV/EHU), San Sebastián, 20018, Spain*

¶*Faculty of Construction and Environmental Engineering, Warsaw University of Life Sciences, 02-776 Warsaw, Poland*

§*Nanospectroscopy Group, Nano-Institute Munich, Department of Physics, Ludwig-Maximilians-Universität München (LMU), Munich 80539, Germany*

‖*Centro de Física de Materiales (MPC, CSIC-UPV/EHU) and Donostia International Physics Center (DIPC), San Sebastián, 20018, Spain*

⊥*Ikerbasque, Basque Foundation for Science, Bilbao, 48013, Spain*

E-mail: yury.rakovich@ehu.eus

Coherence length and pH influence.

The pH influence in J-aggregates formation.

The J-aggregates of JC1 molecules are originated by the pH increment of the aqueous solution. To change the solution pH we have used NaOH in water. Also, the NaOH was added in two different ways: steps of 20 μL every two minutes, or a unique addition. We have chosen two NaOH doses according to the JC1 aqueous solution pH value: 440 μL (11.3) and 620 μL (11.43). A spectra was taken every two minutes to monitor the J-aggregates evolution in time.

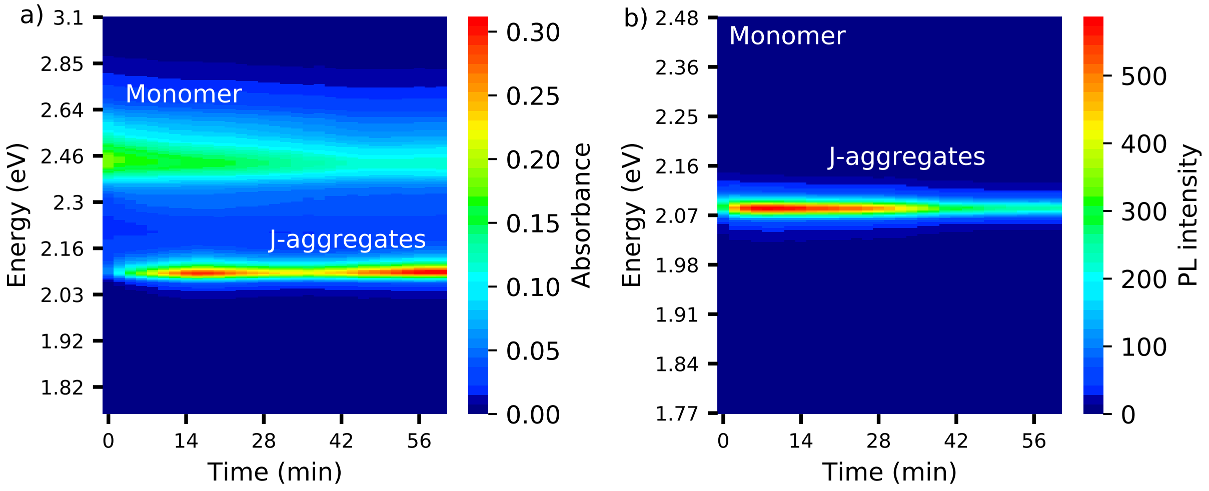

Figure S1: JC1 J-aggregates formation: a) absorbance and b) emission. The 440 μL NaOH put in the solution in 1 addition, and the spectra were taken every 2 minutes. The pH value of the solution was 11.3. In this case, the J-band absorbance response grows quickly. The monomer band tends to diminish at the same time, and it is weaker from the first measure. On the other hand, the J-band photoluminescence is notable from the first measure, and the monomer emission response cannot be appreciable.

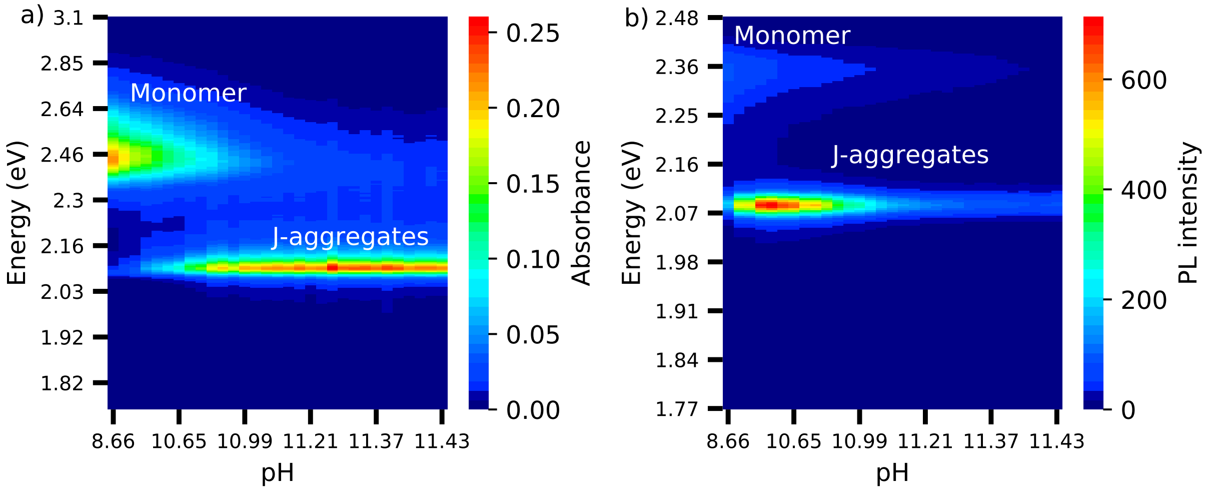

Figure S2: JC1 J-aggregates formation: a) absorbance and b) emission. The 620 μL NaOH were poured in 31 steps. The pH value of the solution increments until 11.43. The J-band absorbance response grows with pH value, reaching its maxima at pH = 11.26. The monomer band tends to diminish at the same time, and it is weaker from the first measure. On the other hand, the J-band photoluminescence is notable from the first measure, and the monomer emission response cannot be appreciable.

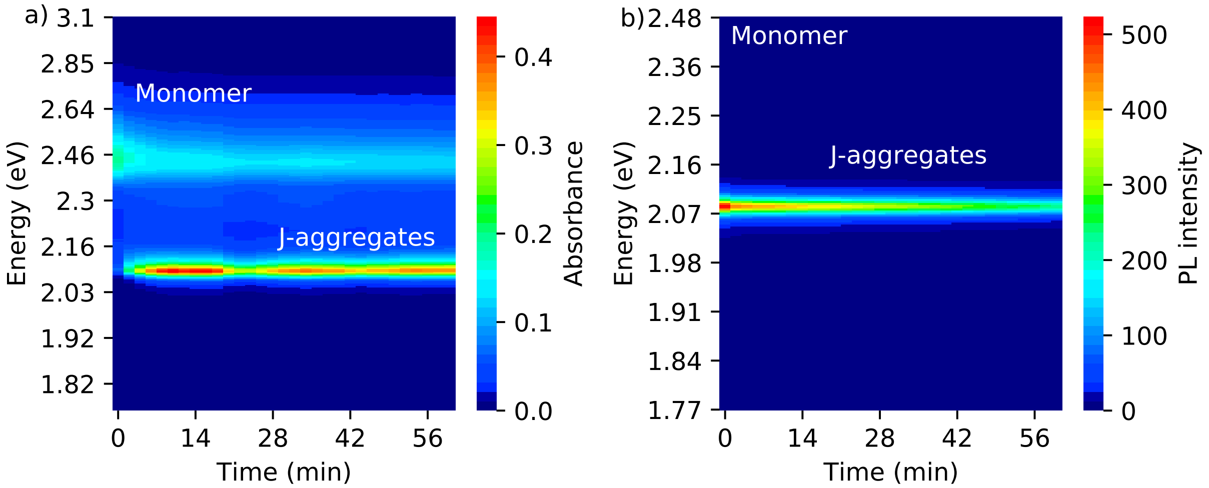

Figure S3: JC1 J-aggregates formation: a) absorbance and b) emission. The 620 μL NaOH were put in the solution in 1 addition, and the spectra were taken every 2 minutes. The pH value of the solution was 11.43. In this case, the J-band absorbance response grows quickly. The monomer band tends to diminish at the same time, and it is weaker from the first measure. On the other hand, the J-band photoluminescence is notable from the first measure, and the monomer emission response cannot be appreciable.

Figures S1, S2, S3 show the J-aggregates formation in water solution in presence of NaOH. For the one-add cases, the evolution is showed in function of time.

Quantum Yield Estimation

JC1 monomer quantum yield was estimated by photon emission comparison with Rhodamine 6G ($\Phi = 0.95$). To obtain comparable solutions, the absorbance spectrum of both dyes must have the same intensity at the excitation wavelength (laser 485 nm). This is shown in Figure S4.

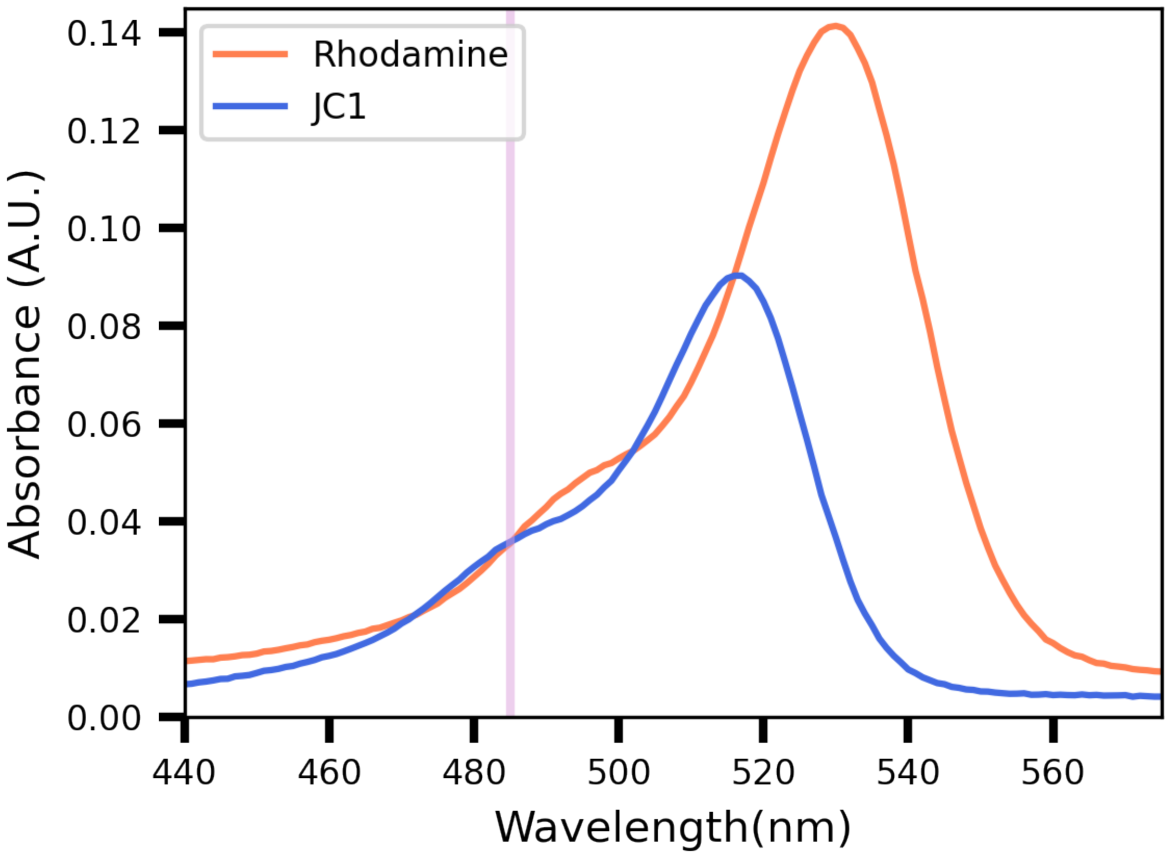

Figure S4: JC1 (blue) and Rhodamine 6G (orange) absorbance spectra in ethanol. The light purple line shows the used laser in PL measurements for sample's excitation at 485 nm.

Then, PL decay measurement of both solutions was performed. The area under the curve represents the emitted photon, we proceeded to integrate both spectra to quantify them.

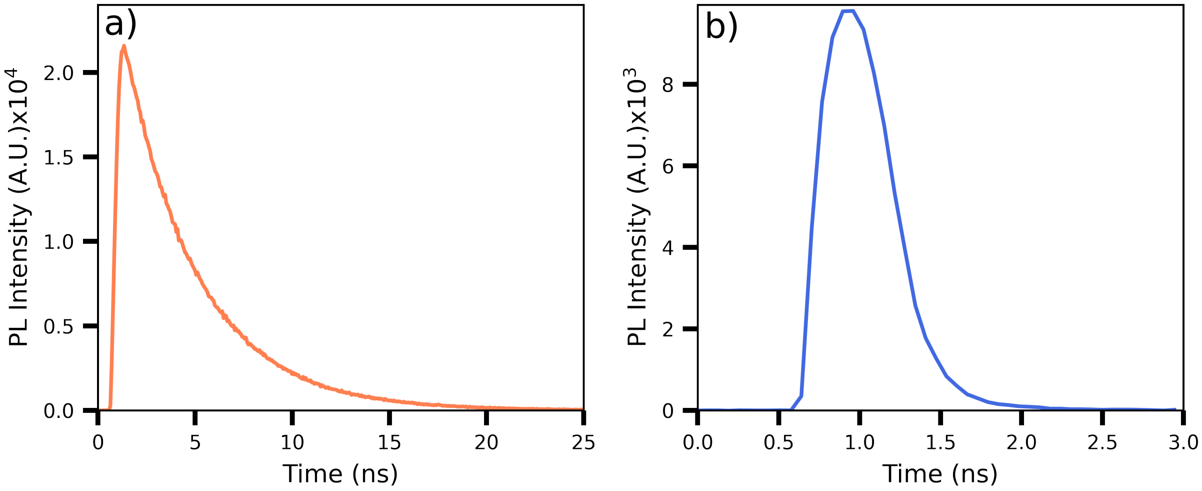

Figure S5: Photoluminescence decay measurement of a) Rhodamine 6G (orange) and b) JC1 monomer (blue) for photon emission count.

The Quantum Yield is defined as the relation between the absorbed and emitted photons. Since both PL measurements were performed under the same conditions and absorbance is equal at 485 nm, we can assure the absorbed photons quantity is the same in both cases and it is possible to relate $\Phi_{Rh6G} = 0.95$ and Φ_{JC1} and estimate the value for $\Phi_{JC1} = 0.05$. To calculate J-aggregates' QY we used Rhodamine 6G and a solution of core (CdSe)-shell (ZnS/CdS/ZnS) quantum dots. We applied the following equation to find the QY value using a reference sample:[1]

$$\Phi_{f,st} = \Phi_{f,st} \frac{F_x}{F_{st}} \frac{f_x(\lambda_{exc})}{f_{st}(\lambda_{exc})} \frac{n_x^2}{n_{st}^2} \tag{S1}$$

Where F is the spectrally integrated photon flux given by the integrated area under the emission spectra, f represents the absorption factor and n the refractive index of the solvent.[1] The 'st' sub index refers to the standard solution with known QY.

To estimate F, we used the following equation:[1]

$$F = \frac{1}{hc_0} \int_{\lambda_1}^{\lambda_2} I_C(\lambda_{exc}, \lambda_{em}) \lambda_{em} d\lambda_{em}) \tag{S2}$$

Where A represents the absorbance value at the excitation wavelength. The Rhodamine 6G was in ethanol solution (n = 1.36), QDs were in hexane solution (n = 1.37) and J-aggregates in water (n = 1.33). The absorbance spectra of the solutions (Figure S6) were used to determine the parameter $A(\lambda_{exc})$, in the case of QDs' QY calculation the excitation wavelength was 490 nm and 580 nm for J-aggregates' QY.

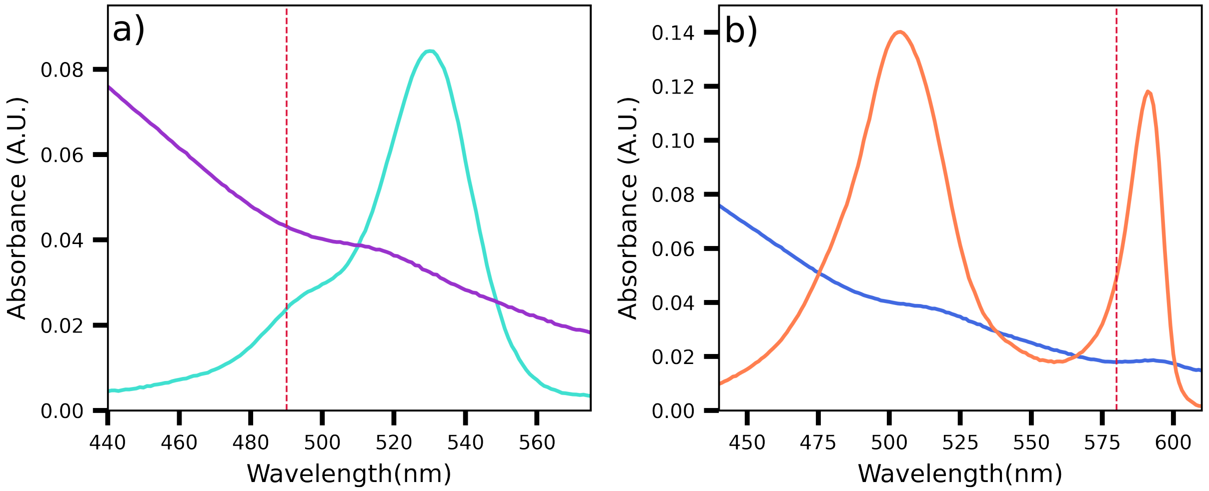

Figure S6: Absorption spectrum of Rhodamine 6G, QDs and J-aggregates. Part a) displays the Rhodamine 6G (light blue) and QDs (violet) absorbance response and the red dotted line shows the excitation wavelength at 490 nm. b) Absorbance spectra of J-aggregates and Quantum Dots. Dashed vertical line indicates excitation wavelength at 580 nm.

Once we established the excitation wavelength we measured the PL spectra to estimate parameter F (equation 2) and then, to apply equation (1). First, we calculated $\Phi_{QDs} = 0.4$, and then Φ_{J-agg}=0.3.

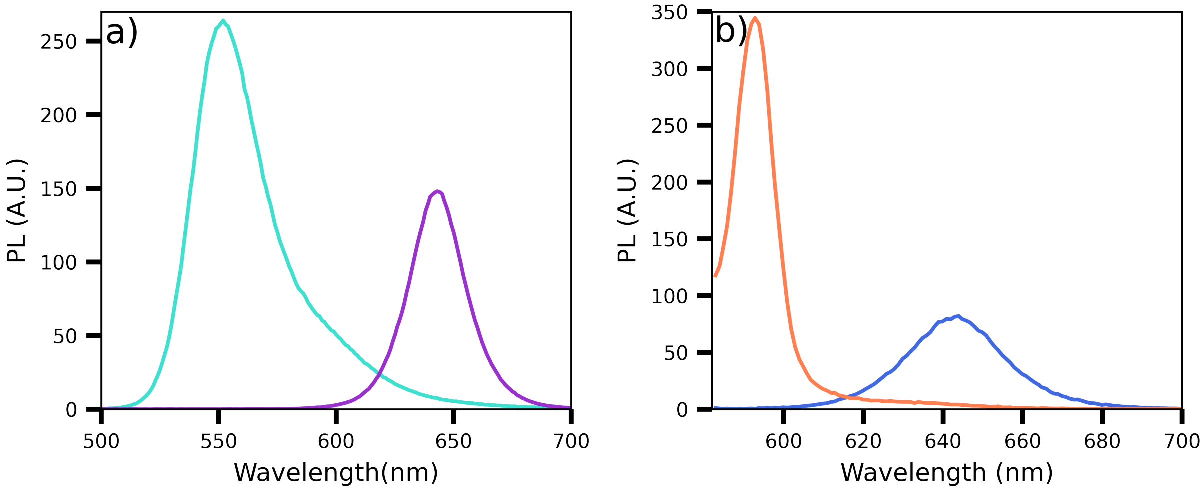

Figure S7: Photoluminescence spectra of Rhodamine 6G, QDs and J-aggregates. a) Rhodamine 6G (light blue) and QDs (violet) PL response with the excitation wavelength at 490 nm. b) Photoluminescence spectra (excitation at 580 nm) of J-aggregates (orange) and Quantum Dots (blue).

Exciton coherence length

The four experiments established to analyze the pH influence in J-aggregates were studied using three different spectroscopic techniques. We estimated the value of the exciton coherence length (N_{coh}) using each of them. Two of them were analysed at constant pH (NaOH added in one dose, 440 μL with pH= 11.3 and 620 μL with pH= 11.45). The value of N_{del} is presented in function of time.

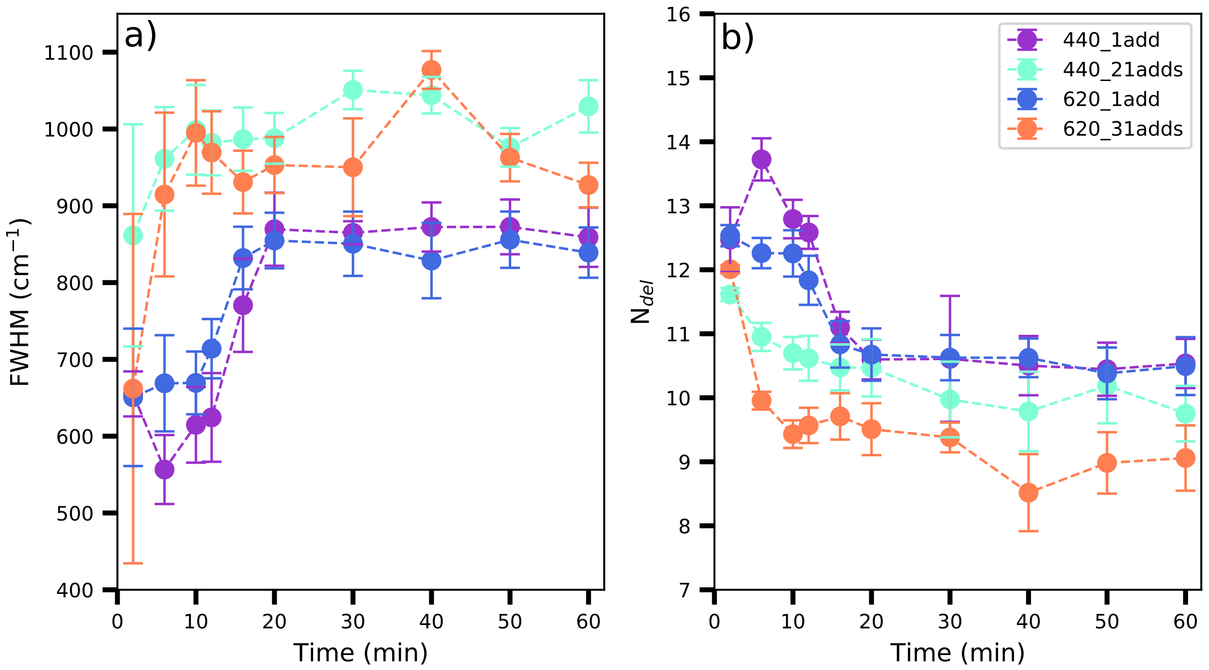

Figure S8: Fitted FWHM and estimated coherence lenght N_{coh} for each pH experiment. a) The FWHM parameter was obtained from Voigt fitting for each absorbance spectrum in each case. b) Calculated N_{coh} from estimated FWHM.

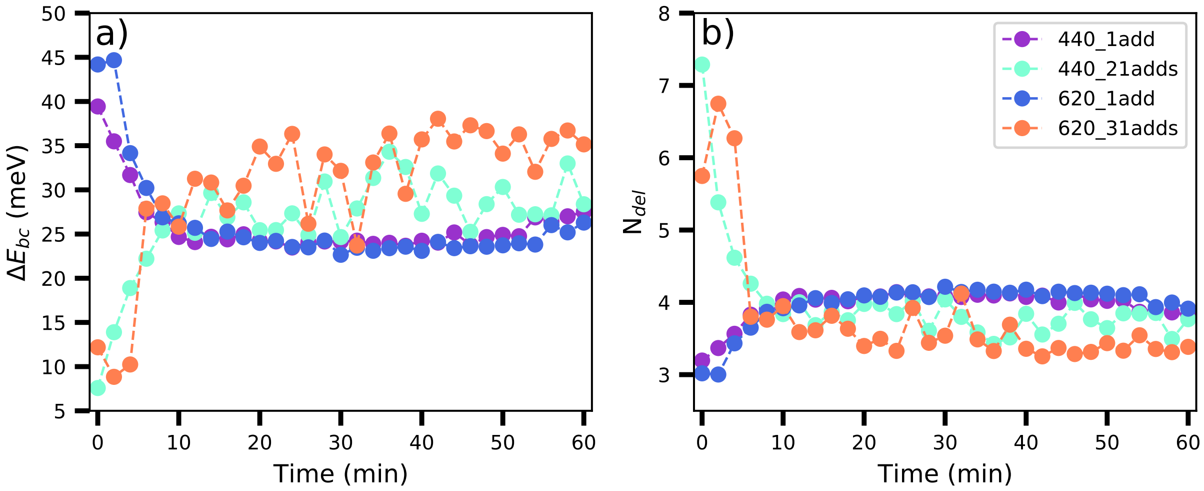

Figure S9: Barycenters difference and exciton coherence lenght. a) Difference between emission and absorbance barycenters (ΔE_{bc}) and b) estimated N_{coh} for each pH experiment.

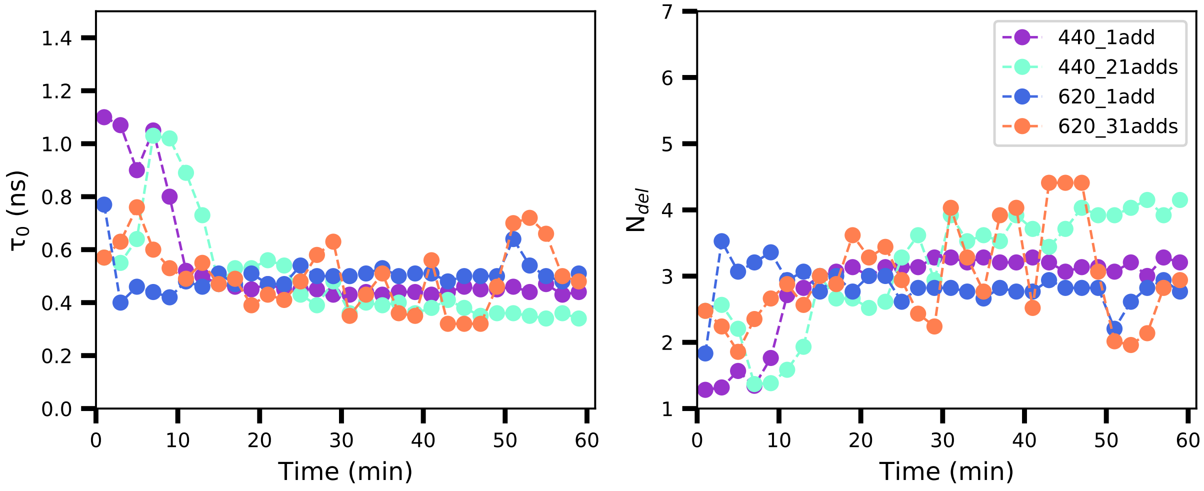

Figure S10: Lifetime and estimated coherence lenght N_{coh} for each pH experiment. a) Measured lifetime using MicroTime of J-aggregates evolution, each measure was taken every two minutes. b) Calculated N_{coh} from lifetime.

Monomer and J-aggregates mean lifetime estimation

The maximum possible mean lifetime (τ_0) of monomer and J-aggregates was estimated by the analysis of PL intensity decay, and by taking the pH variation into account. By using the equation derived from Einstein's fundamental relationship of the transition probabilities for induced absorption and spontaneous emission,[2] we are able to determine τ_0.

$$\frac{1}{\tau_0} = A_{u\to l} = 8 \times 2303\pi c\tilde{\nu}_{ul}^2 n^2 \mathfrak{N}^{-1} \frac{g_l}{g_u} \int \epsilon d\tilde{\nu} \tag{S3}$$

The Einstein transition probability coefficient for spontaneous emission from state u to l gives the inverse of the mean lifetime (τ_0).[2] This constant rate is related to the speed of light c, the frequency of transmission $\tilde{\nu}_{ul}$ (cm^{-1}), the refraction index n, Avogadro's number $\mathfrak{N}$, the relation of the degeneracies of the implied states g_l and g_u (was assumed to be 1), and the integral of the molar extinction ϵ. Equation S3 is defined for the case when absorption and emission occur at the same wavelength, otherwise we have $\tilde{\nu}_{ul}^2 = \langle \nu_f^{-3} \rangle_{AV}^{-1}$, and it can be calculated from the spectrum's intensity $I(\nu)$.[2]

$$\langle \nu_f^{-3} \rangle_{AV}^{-1} = \frac{\int I(\nu) d\nu}{\int \nu^{-3} I(\nu) d\nu} \tag{S4}$$

The obtained J-aggregates' τ_0^M using Equation S3 is 2.6 ns (in average), and for the monomeric response $\tau_0^M = 0.7$ ns. The estimated τ_0 from Equation S3 represents the pure radiative lifetime. It may be equal to the average lifetime obtained from experimental PL decay curves in the ideal case, when the quantum yield value is 1.

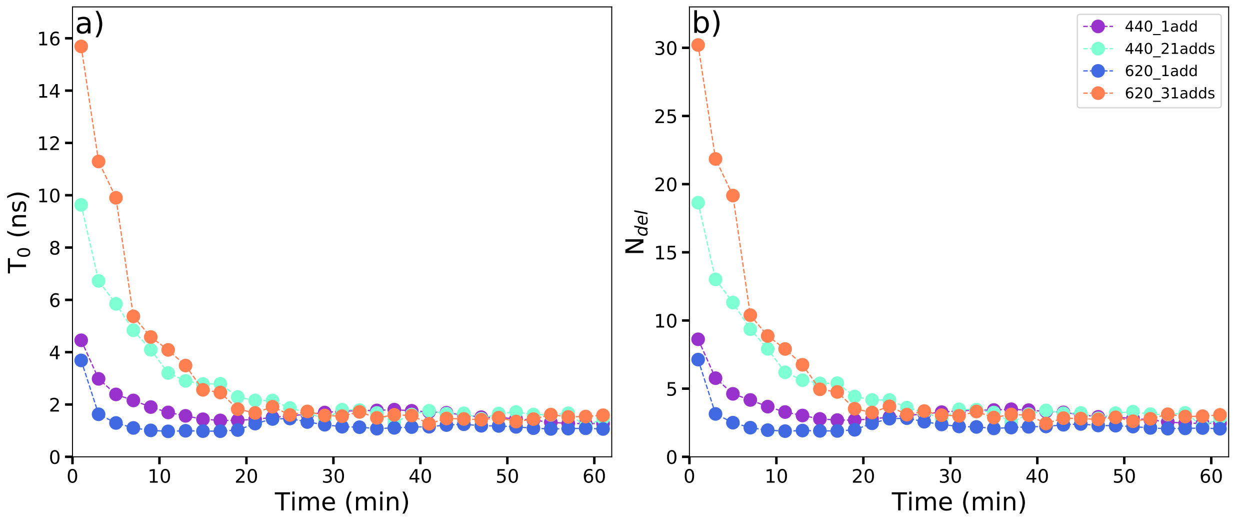

Figure S11: Calculated mean lifetime (τ_0) and exciton coherence lenght N_{coh}. a) The estimated τ_0 obtained using equation S3 and b) coherence lenght for each experiment.

The measured average lifetime (τ) of J-aggregates is 0.5 ns (τ_0^J = 1.8 ns) and 0.18 ns (τ_0^M = 3.2 ns) for monomeric PL (in average). In the case of J-aggregates, the estimated radiative lifetime from Equation S3 is 1.4 times shorter than the observed one. It must be pointed out that we consider molecular chains response instead of a non-aggregated molecule response. However, the monomer estimated τ_0 and the proximity to the equipment's resolution limit of the measured τ (0.15 ns) suggest a short monomer lifetime value. Also, the non-homogeneous shape of the spectra (the monomer and J-band peaks are non-symmetric) can be associated with a certain degree of error in the indicated values. This aspect is usually associated with vibrational coupling in molecules[3] and disorder in the system. Moreover, the strong influence of experimental excitation intensity on the decay rate[4] must be taken into account, because

the PL spectra and lifetimes were measured using different equipment.

Reported coherence length

The value of N_{coh} has been reported for several J-aggregates of cyanine dyes, these values were obtained from different experimental analysis like the monomer and aggregates concentration comparison (using absorbance spectra), enthalpy consideration, transient absorption spectroscopy, etc. The coherence length of each one of these dyes and the used technique is summarized in the following table.

Table S1: Table with the values of N_{coh} reported for J-aggregates of certain compounds.

Compound	N_{coh}	Experimental Technique	Reference
BIC	12-13	PL decay, T = 60 K	[5]
Cyanine dye S120	30	TEM	[6]
Cy3	55	Absorption linewidth	[7]
C8O3 tubular TDBC	95	Transient absorption, T = 1.5 K	[8]
DMDC	4	Absorption (water)	[9]
JC1	8	2-D Aggregation structure	[10]
	30	Absorption, RT	[11]
L-21	32	Absorption	[12]
MC	10	Absorption	[6]
Me-DS	26	Absorption, transient absorption	[13]
amphi-PIC	30	Absorption	[14]
PIC	2-3	Absorption	[15]
	4	PL decay	[16]
	5	Radiative lifetime, T = 140K	[17]
	5-29	Pressure-induced absorption	[18]
	7-25	Ethalpy consideration	[19]
	8.9	Relative PL intensities	[20]
	26	Absorption, RT	[21]
	30	Absorbance linewidth	[22]
	60	Absorption	[23]
	70	Two-color transient absorption	[24]
	100	Transient absorption	[25]
	100	PL decay, T = 4.2 K	[26]
	100	Transient absorption	[27]
PIC-AgBr	2.1-2.5	Absorption, RT	[28]
PIC-Br	10	PL decay, T = 220 K	
	20	Transient absorption	[29]
	50	Absorption, T = 1.5 K	[30]
	115	PL decay, T = 1.5 K	[31]
	340	Transient absorption, T = 77 K	[32]
PIC-I	100	Absorption, PL at T = 77 K	[33]
TC	4	Absorption linewidth	[34]
TDC	4	Absorption linewidth	[34]
	25	Absorption linewidth	[35]
TDBC	4	Monomers and aggregates concentration comparison.	[36]
	6	PL decay	[37]
	6.3, 6.3, 7.9, 8.3, and 9.4	Radiative rates (different surfactants)	[38]
	10	PL lifetime	[39]

**Compound**	**N$_{coh}$**	**Experimental Technique**	**Reference**
TDBC (C2S4)	16	Femtosecond spectroscopy, RT	[4]
	30 - 45	Transient absorption, T = 1.5 K	[40]
THIATS	9	Absorption linewidth	[41]
	17	Radiative lifetime, T= 140K	[17]
	19	Absorbance linewidth	[35]
Thyacarbocyanine	6	Absorption	[42]

References

(1) Grabolle, M.; Spieles, M.; Lesnyak, V.; Gaponik, N.; Eychmüller, A.; Resch-Genger, U. Determination of the fluorescence quantum yield of quantum dots: suitable procedures and achievable uncertainties. *Anal. Chem.* **2009**, *81*, 6285–6294.

(2) Strickler, S. J.; Berg, R. A. Relationship between absorption intensity and fluorescence lifetime of molecules. *J. Chem. Phys.* **1962**, *37*, 814–822.

(3) de Jong, M.; Seijo, L.; Meijerink, A.; Rabouw, F. T. Resolving the ambiguity in the relation between Stokes shift and Huang–Rhys parameter. *Phys. Chem. Chem. Phys.* **2015**, *17*, 16959–16969.

(4) van Burgel, D. A., M.; Wiersma; Duppen, K. The dynamics of one-dimensional excitons in liquids. *J. Chem. Phys.* **1995**, *102*, 20–33.

(5) Kamalov, V. F.; Struganova, I. A.; Tani, T.; Yoshihara, K. Temperature dependence of superradiant emission of BIC J-aggregates. *Chem. Phys. Lett.* **1994**, *220*, 257–261.

(6) Nakahara, H.; Fukuda, K.; Moebius, D.; Kuhn, H. Two-dimensional arrangement of chromophores in J aggregates of long-chain merocyanines and its effect on energy transfer in monolayer systems. *J. Phys. Chem.* **1986**, *90*, 6144–6148.

(7) Thomas, R.; Thomas, A.; Pullanchery, S.; Joseph, L.; Somasundaran, S. M.; Swathi, R. S.; Gray, S. K.; Thomas, K. G. Plexcitons: the role of oscillator strengths and spectral widths in determining strong coupling. *ACS Nano* **2018**, *12*, 402–415.

(8) Lampoura, S. S.; Spitz, C.; Dähne, S.; Knoester, J.; Duppen, K. The optical dynamics of excitons in cylindrical J-aggregates. *J. Phys. Chem. B* **2002**, *106*, 3103–3111.

(9) Ballard, R. E.; Gardner, B. J. The J-band of 1,1'-diethyl-9-methyl-4,5;4',5'-dibenzthiacarbocyanine chloride. *J. Chem. Soc. B* **1971**, 736–738.

(10) Sato, N.; Fujimura, T.; Shimada, T.; Tani, T.; Takagi, S. J-aggregate formation behavior of a cationic cyanine dye on inorganic layered material. *Tetrahedron Lett.* **2015**, *56*, 2902–2905.

(11) Savateeva, D.; Melnikau, D.; Lesnyak, V.; Gaponik, N.; Rakovich, Y. P. Hybrid organic/inorganic semiconductor nanostructures with highly efficient energy transfer. *J. Mater. Chem.* **2012**, *22*, 10816–10820.

(12) Guralchuk, G. Y.; A. V. Sorokin, I. K. K.; Yefimova, S. L.; Lebedenko, A. N.; Malyukin, Y. V.; Yarmoluk, S. M. Specificity of cyanine dye L-21 aggregation in solutions with nucleic acids. *J. Fluoresc.* **2007**, *17*, 370–376.

(13) Duschl, C.; Frey, W.; Knoll, W. The crystalline structure of two-dimensional cyanine dye single crystals as revealed by electron diffraction. *Thin Solid Films* **1988**, *160*, 251–255.

(14) Yefimova, S.; Sorokin, A.; Katrunov, I.; Malyukin, Y. Exciton localization effects in nanoscale molecular clusters (J-aggregates). *Low Temp. Phys.* **2011**, *37*.

(15) Zimmermann, H.; Scheibe, G. Zur Konstitution und Lichtabsorption der reversibel polymeren Form des Pseudoisocyanins. *Z. Elektrochem.* **1956**, *60*, 566–569.

(16) Michelbacher, E. Abklingzeitmessungen an Pseudoisocyanindiäthylchlorid mit einem Phasenfluorometer mit 200 MHz-Lichtmodulation:. *Z. Naturforsch. A* **1969**, *24*, 790–796.

(17) Scheblykin, I.; Bataiev, M.; Van der Auweraer, M.; Vitukhnovsky, A. Dimensionality and temperature dependence of the radiative lifetime of J-aggregates with Davydov splitting of the exciton band. *Chemical Phys. Lett.* **2000**, *316*, 37–44.

(18) Neumann, B.; Pollmann, P. Aggregation of pseudoisocyanine chloride in aqueous solutions at high pressures. *Ber. Bunsenges. Phys. Chem.* **1996**, *100*, 15–19.

(19) Daltrozzo, E.; Scheibe, G.; Gschwind, K.; Haimerl, F. Structure of the J-aggregates of pseudocyanine. *Photogr. Sci. Eng.* **1974**, *18*, 441–450.

(20) WP, B.; JL, B.; MB, S.; M., B. Molecular model of J-aggregated pseudoisocyanine fibers. *J. Chem. Phys.* **2018**, *149*.

(21) Melnikau, D.; Savateeva, D.; Chuvilin, A.; Hillenbrand, R.; Rakovich, Y. P. Whispering gallery mode resonators with J-aggregates. *Opt. Express* **2011**, *19*, 22280–22291.

(22) Fukutake, N.; Kobayashi, T. Size distribution of pseudoisocyanine (PIC) J-aggregates studied by near-field absorption spectroscopy. *Chem. Phys. Lett.* **2002**, *356*, 368–374.

(23) Knapp, E. Lineshapes of molecular aggregates, exchange narrowing and intersite correlation. *Chem. Phys.* **1984**, *85*, 73–82.

(24) Durrant, J. R.; Knoester, J.; Wiersma, D. A. Local energetic disorder in molecular aggregates probed by the one-exciton to two-exciton transition. *Chem. Phys. Lett.* **1994**, *222*, 450–456.

(25) Kopainsky, B.; Kaiser, W. Ultrafast transient processes of monomers, dimers, and aggregates of pseudoisocyanine chloride (PIC). *Chem. Phys. Lett.* **1982**, *88*, 357–361.

(26) Fidder, H.; Terpstra, J.; Wiersma, D. A. Dynamics of Frenkel excitons in disordered molecular aggregates. *J. Chem. Phys.* **1991**, *94*, 6895–6907.

(27) R. Gagel, R. G.; Laubereau, A. Evidence for biexcitons and dynamic Stark effect in J-aggregates from femtosecond spectroscopy. *Chem. Phys. Lett.* **1994**, *217*, 228–233.

(28) Lanzafame, J. M.; Muenter, A. A.; Brumbaugh, D. V. The effect of J-aggregate size on photoinduced charge transfer processes for dye-sensitized silver halides. *Chem. Phys.* **1996**, *210*, 79–89.

(29) Minoshima, K.; Taiji, M.; Misawa, K.; Kobayashi, T. Femtosecond nonlinear optical dynamics of excitons in J-aggregates. *Chem. Phys. Lett.* **1994**, *218*, 67–72.

(30) Fidder, H.; Knoester, J.; Wiersma, D. A. Observation of the one-exciton to two-exciton transition in a J aggregate. *J. Chem. Phys.* **1993**, *98*, 6564–6566.

(31) De Boer, S.; Wiersma, D. A. Dephasing-induced damping of superradiant emission in J-aggregates. *Chem. Phys. Lett.* **1990**, *165*, 45–53.

(32) Kobayashi, S.; Sasaki, F. Ultrafast spectroscopy of PICBr J aggregates: the dynamics of large coherence length exciton. *J. Lumin.* **1994**, *58*, 113–116.

(33) Hamanaka, Y.; Kurasawa, H.; Nakamura, A.; Uchiyama, Y.; Marumoto, K.; Kuroda, S. Femtosecond transient absorption study of merocyanine J-aggregates. *J. Lumin.* **2001**, *94-95*, 451–455.

(34) Sorokin, A. V.; Zabolotskii, A. A.; Pereverzev, N. V.; Yefimova, S. L.; Malyukin, Y. V.; Plekhanov, A. I. Plasmon controlled exciton fluorescence of molecular aggregates. *J. Phys. Chem. C* **2014**, *118*, 7599–7605.

(35) Vranken, N.; Van der Auweraer, M.; De Schryver, F. C.; Lavoie, H.; Bélanger, P.; Salesse, C. Influence of molecular structure on the aggregating properties of Thiacarbocyanine dyes adsorbed to langmuir films at the air-water interface. *Langmuir* **2000**, *16*, 9518–9526.

(36) Herz, A. Aggregation of sensitizing dyes in solution and their adsorption onto silver halides. *Adv. Colloid Interface Sci.* **1977**, *8*, 237–298.

(37) Spitz, C.; Daehne, S. Architecture of J-aggregates studied by pressure-dependent absorption and fluorescence measurements. *Ber. Bunsenges. Phys. Chem.* **1998**, *102*, 738–744.

(38) De Rossi, U.; Daehne, S.; Lindrum, M. Increased coupling size in J-aggregates through N-n-Alkyl betaine surfactants. *Langmuir* **1996**, *12*, 1159–1165.

(39) Anantharaman, S. B.; Kohlbrecher, J.; Rainò, G.; Yakunin, S.; Stöferle, T.; Patel, J.; Kovalenko, M.; Mahrt, R. F.; Nüesch, F. A.; Heier, J. Enhanced Room-Temperature Photoluminescence Quantum Yield in Morphology Controlled J-Aggregates. *Adv. Sci.* **2021**, *8*, 1903080.

(40) J. Moll, J. R. D., S.Daehne; Wiersma, D. A. Optical dynamics of excitons in J aggregates of a carbocyanine dye. *J. Chem. Phys.* **1995**, *102*, 6362–6370.

(41) Rousseau, E.; Van der Auweraer, M.; De Schryver, F. C. Steady-state and time-resolved spectroscopy of a self-assembled cyanine dye multilayer. *Langmuir* **2000**, *16*, 8865–8870.

(42) Ivanov, A. A.; D. A. Akimov, P. V. M.; Plekhanov, A. I.; Alfimov, M. V.; Zheltikov, A. M. Pump-probe nonlinear absorption spectroscopy of molecular aggregates using chirped frequency-shifted light pulses from a photonic-crystal fiber. *Laser Phys.* **2006**, *16*, 965–969.
