# Supplementary material for: Cross Determination of Exciton Coherence Length in J-Aggregates
Source: J Phys Chem Lett. 2022 Oct 25;13(43):10198–206. doi: 10.1021/acs.jpclett.2c02213 (PMC10401724; doi:10.1021/acs.jpclett.2c02213)
Supplement: Supplementary file 2 — jz2c02213_si_002.pdf [file jz2c02213_si_002.pdf]

jz-2022-022136.R1

Name: Peer Review Information for "Cross Determination of Exciton Coherence Length in J-aggregates"

First Round of Reviewer Comments

Reviewer: 1

Comments to the Author

The manuscript describes estimate of the coherence length of J-aggregates using different methods in order to clarify which method is the most appropriate. The work has sense, but in case if a reference method to determine the “real” coherence length is available, for example, via simulation. Using four methods, which give rather large scattering of the results, i.e., from 2 to 10, it is difficult to make a solid conclusion concerning the choice in favor of the correctness of the method. Moreover, the manuscript represents a kind of technical work with no explanations of physical background of each method for determining the coherence length and which restrictions exist for them. The central point is that the exciton delocalization increases with suppression of exciton-vibrational coupling, which is clearly related to narrowing the absorption bandwidth and decreasing the Stokes shift, justifying the first two methods. However, the methods based on radiative lifetimes and transient absorption spectroscopy, need additional justification. For example, Eq. (6) can be wrong in cases where the monomer emission is accompanied by strong molecular twisting which leads to abnormal reduction in radiative lifetime of the monomer. Restrictions of Eq. (8) were discussed in Refs. 16 and 17.

The other drawback of the manuscript is that the authors missed other well-known method for estimate of the coherence length based on comparison of the 0-0 and 0-1 emission bands, i.e., the intensity ratio of the main electronic transition to the first vibronic sideband in the low-temperature PL spectrum (see the work of Spano , Acc. Chem. Res. 2010, 43, 429−439.).

Minor remarks:

1. It is not clear the estimate of Ncoh based on Eqs. (4) and (5). The ratio of ∆Ebc of the monomer and J-aggregate is 5, and Ncoh from above Eqs. is ~ 2, not 4, as stated in the manuscript. Please explain.

2. It is stated in the abstract that the coherence length defines long-range exciton transport, which should be explained. Normally, Forster exciton energy transfer is dependent on donor-acceptor coupling (distance), spectral overlap of emission/absorption spectra, and exciton-phonon coupling. The above statement can be related only to the latter.

3. In the abstract, it should be specified the molecules for which the values of the coherence length are given, which is not the universal number of course.

4. Experimental details and measurement of the PL quantum yield should be given in SI.

In conclusion, after the major revision the manuscript could be appropriate for more specialized journal.

Reviewer: 2

Comments to the Author

The manuscript reports on various methods to determine the coherence length in isocyanine J-aggregates, highlighting the various methods used and the variation in the coherence numbers obtained. It is clearly written and the subject is very timely, since the coherence length of excitons plays a very important role in energy transport for applications such as solar cells. Coherence is also a central topic in quantum computing. The paper should be published subject to a successful response to the following:

1) In eq.(2) is the coupling J an effective nearest-neighbor coupling?

2) Eq.(4) is approximate in that it assumes the HR factor is sufficiently large that the absorption and emission spectra can be modelled with Gaussian line shapes (with unresolved vibronic peaks). This may be worth a mention since the monomer HR factor from Fig 1a is quite small - maybe 0.3.

3) There is a more recent method based on HR factors for determining the coherence number. Based on the monomer HR factor Smon (for the vinyl stretching mode -around 0.18 eV) which can readily be determined from the spectra in Fig.1a, the coherence number is given by

Ncoh = { I0-0 / I0-1} * Smon

where I0-0 is the line strength of the 0-0 transition in the J-aggregate PL spectrum and I0-1 is line strength of the first side-band in the PL spectrum. (I0-0 and I0-1 refer to the peak intensities in a spectrum where the cubic dependence on the photon frequency has been deconvoluted away). Details appear in,

1) Spano, FC and Yamagata, H

May 12 2011 | JOURNAL OF PHYSICAL CHEMISTRY B 115 (18) , pp.5133-5143

2) Hestand, NJ and Spano, FC

Aug 8 2018 | CHEMICAL REVIEWS 118 (15) , pp.7069-7163

The authors should at least mention this method.

Author's Response to Peer Review Comments:

Dear Dr. Editor,

We would like to thank the reviewers for helping us to improve our manuscript with their comments. We found their assessment overall positive and valuable. Considering all the points enlightened by the reviewers, we proceed to enforce our manuscript and develop a revised version. A point-by-point response to the reviewer's comments is provided in the attached document.

Prof. Yury Rakovich
Ikerbasque Research Professor
Materials Physics Center
Paseo Manuel Lardizábal 5
Donostia-San Sebastián, Spain

September 22, 2022

Manuscript ID: jz-2022-022136

Prof. Editor
Senior Editor
The Journal of Physical Chemistry Letters

Dear Dr. Editor,

We would like to thank the reviewers for helping us to improve our manuscript with their comments. We found their assessment overall positive and valuable. Considering all the points enlightened by the reviewers, we proceed to enforce our manuscript and develop a revised version. We hope that our improvements and explanations will be found complete and satisfactory.

Yury Rakovich

Replies to editorial comments:

1. Please label as TOC GRAPHIC.

   We have modified the label of TOC graphic.

2. Remove the section heading(s) throughout the manuscript (but leave "Abstract" and "TOC Graphic").

   The headlines have been removed.

3. In both the main file and the supporting information, fix the style of all references to use JPCL formatting (check all references carefully).

   The style of the references has been changed accordingly.

4. Please label equations as S1, S2, etc.

   We have changed labels of all equations in supporting information file.

5. Please label tables as Table S1, Table S2, etc.

   We have changed labels of all tables in the supporting information file.

Replies to reviewers:

Reviewer #1

1. The manuscript describes estimate of the coherence length of J-aggregates using different methods in order to clarify which method is the most appropriate. The work has sense, but in case if a reference method to determine the "real" coherence length is available, for example, via simulation. Using four methods, which give rather large scattering of the results, i.e., from 2 to 10, it is difficult to make a solid conclusion concerning the choice in favor of the correctness of the method. Moreover, the manuscript represents a kind of technical work with no explanations of physical background of each method for determining the coherence length and which restrictions exist for them. The central point is that the exciton delocalization increases with suppression of exciton-vibrational coupling, which is clearly related to narrowing the absorption bandwidth and decreasing the Stokes shift, justifying the first two methods. However, the methods based on radiative lifetimes and transient absorption spectroscopy, need additional justification. For example, Eq. (6) can be wrong in cases where the monomer emission is accompanied by strong molecular twisting which leads to abnormal reduction in radiative lifetime of the monomer. Restrictions of Eq. (8) were discussed in Refs. 16 and 17.

   We thank the reviewer for discussing about the radiative lifetimes and transient absorption spectroscopy methods' limitations. Following the discussion of radiative rates, we would like to remark that according to the seminal work of Kasha (29, 30), the dipole moment of a linear chain of molecules (i.e., J-aggregates) increases as $\mu_J = (N_{coh})^{1/2}\ \mu_J$, where μ_J and μ_{mon} are the dipole moments of the aggregate and monomer, respectively. Since the radiative rate constant for the dipole-allowed transition can be expressed as $k_{rad} = 4\mu^2 /3\hbar\lambda^3 c^3$, N_{coh} can be found using radiative rate constants of the exciton and monomer based on the measurement of emission decay. The applicability of Equation (6) can be limited by warping that in turns can affect the molecules in general. Such deformation can oppose the delocalization of exciton (27).

   As it is stablished in this comment, for each method we confront with some applicability limits, and we included in our manuscript the corresponding justifications.

   We added the following sentence on page 7:

   > "However, this approach for estimating N_{coh} was not seen as too accurate and precise because the bandwidth of the monomers forming J-aggregates may differ from that of monomers (20, 21)".

   Also, the following text has been added (page 8):

   > "The justification for Equation 1 arises from the increase in N_{coh} with the suppression of exciton-vibrational coupling and hence with a narrowing of the absorption band. However, the use of equation (1) must be taken with caution because the correct measurement of the J value from the absorption shift of the J-band (equation 2) requires accounting for the ground state level shift of the molecules (20)".

   The following text has been added (page 9) as justification for the Huang-Rhys parameter use in N_{coh} calculation:

   > "The Stokes shift is usually calculated as the difference between absorbance and emission maxima and is related to the dimensionless Huang-Rhys (HR) parameter, which is a measure of the electron-phonon or electron–vibrational coupling strength (24). Because

of the ambiguity of the relationship between the Stokes shift and the HR parameter used in different papers, Jong et al. presented a more advanced analysis for the influence of thermal effects in the band maximum position and how to correct for possible errors due to these effects".

Text added to page 10:

"The rationalization of this equation is that the shape of the spectral lines and the linewidth (which in turn is defined by the value of N) depend on the vibrational coupling strength and hence on S (24)".

To justify the equation used for N_{coh} calculation using J-aggregates photoluminescence, we added the following paragraphs to page 11-12:

"According to the seminal work of Kasha (29, 30), the dipole moment of a linear chain of molecules (i.e., J-aggregates) increases as $\mu_J = (N\ coh)^{1/2}\ \mu_J$, where μ_J and μ_{mon} are the dipole moments of the aggregate and monomer, respectively. Since the radiative rate constant for the dipole-allowed transition can be expressed as $k_{rad} = 4\mu^2 / 3\hbar\lambda^3\ c^3$, Ncoh can be found using radiative rate constants of the exciton and monomer based on the measurement of emission decay".

"The applicability of Equation (6) can be limited by warping affecting the molecules in general. Such deformation can oppose the delocalization of exciton (27)".

Text added to page 15, to justify the used equation in transient absorption spectroscopy analysis:

"The limits of equation (8) application are given by effect of saturation: the pump-probe spectrum shape is dominated by homogeneous line width. The origin of this saturation is the overlap of bleaching and induced-absorption contributions due to further disorder decrease (17). Then, Δ is related to the saturation length (16)".

2. The other drawback of the manuscript is that the authors missed other well-known method for estimate of the coherence length based on comparison of the 0-0 and 0-1 emission bands, i.e., the intensity ratio of the main electronic transition to the first vibronic sideband in the low-temperature PL spectrum (see the work of Spano, Acc. Chem. Res. 2010, 43, 429−439.).

We appreciate this comment about this relevant PL related N_{coh} estimation method and the provided references. The presented method relates N_{coh} with temperature and disorder (σ). It is stablished that it is possible to apply the line strength ratio to evaluate exciton coherence length as long as kb.T, $\sigma \ll \omega$ (1). Although this method presents a very reliable way to obtain the exciton coherence length, the study of coherence length on temperature and extent of disorder is beyond the scope of our work and these parameters are not among the variables we consider. We plan to do such research in the future. To clarify this point we have added the following text on page 14:

"There is another method related to PL response and N_{coh}. It is stablished that it is possible to apply the PL line strength ratio to evaluate exciton coherence length at room temperature as long as $k_b .T$, σ(disorder)$\ll \omega$ (34). Although this method presents a very reliable way to obtain the exciton coherence length, the study of coherence length

on temperature and extent of disorder is beyond the scope of our work and these parameters are not among the variables we consider. We plan to do such research in the future".

Also, the corresponding reference was added:

(34) Spano, F. C.; Yamagata, H. Vibronic coupling in J-aggregates and beyond: a direct means of determining the exciton coherence length from the photoluminescence spectrum. *J. Phys. Chem. B* **2011**, *115*, 5133–5143.

Minor remarks:

1. It is not clear the estimate of N_{coh} based on Eqs. (4) and (5). The ratio of ΔE_{bc} of the monomer and J-aggregate is 5, and N_{coh} from above Eqs. is ~ 2, not 4, as stated in the manuscript. Please explain.

We thank reviewer for this comment. The estimation of N_{coh} was reviewed and an error in the applied equation was found. The value of N_{coh} using this method is ~3 in average. Figure 4 b and the corresponding text in the manuscript have been changed as follows:

The following text was added in page 10 to correct the N_{coh} value provided:

"The value of the coherence length obtained from S is 3 on average (Figure 4 b). The value of N_{coh} decreases with J-aggregate concentration. The absorbance spectra reveal the maximal J-aggregate concentration at pH 11.2 (see Figure 2 a). The first value of N_{coh} is the highest (5.2) and the final one (2.6) is reached at the highest pH value (11.3). Conversely, the first ΔE_{bc} value is the lowest (7 meV), increasing up to almost 35 meV. Evolutions of these quantities are shown in Figure 4 b)".

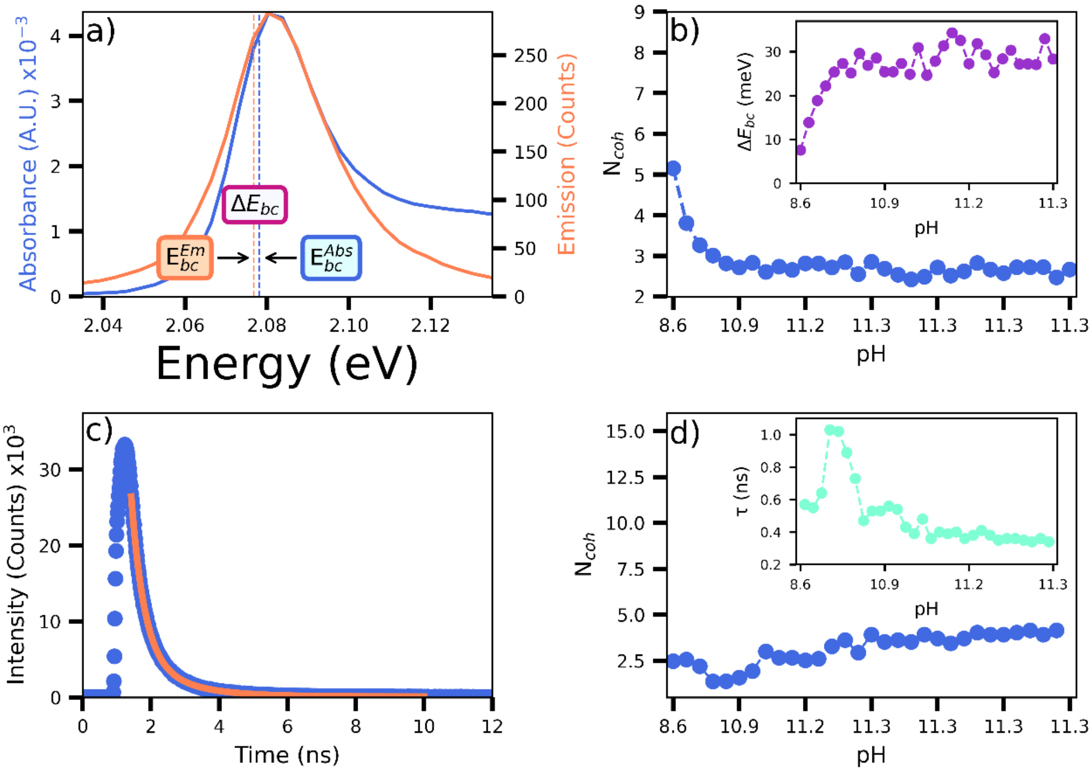

Figure 4.- Results of analysis of J-aggregate absorption and PL to calculate the exciton coherence length: (a) Stokes shift ΔE_{bc} estimation using energy barycenters of absorbance (blue) and emission (orange) spectra, b) evolution of the coherence length (estimated from Huang-Rhys parameter) with pH. c) Photoluminescence intensity decay (blue circles) and the result of the double exponential fitting (orange line) of JC1 solution with pH = 8.6, and d) photoluminescence lifetimes (τ) of J-aggregates (aquamarine dots) and N_{coh} calculated from radiative decay rates of monomer and J-aggregates in fuction of pH.

2. It is stated in the abstract that the coherence length defines long-range exciton transport, which should be explained. Normally, Forster exciton energy transfer is dependent on donor-acceptor coupling (distance), spectral overlap of emission / absorption spectra, and exciton-phonon coupling. The above statement can be related only to the latter.

We are grateful to the reviewer for pointing out the discrepancy in this phrase. Clearly, being a characteristic intrinsic to the exciton as a whole, the coherence length cannot define the motion of the exciton wave packet, i.e. the exciton transport, although it can to some extent determine its character - coherent or incoherent. We have made the relevant changes to the abstract:

"The coherence length of the Frenkel excitons (N_{coh}) is one of the most critical parameters governing many key features of supramolecular J-aggregates".

Also, a new sentence has been added to the introduction section:

"The coherence length also has some implications for the nature of long-distance exciton migration and transport - whether coherent or incoherent (5)".

3. In the abstract, it should be specified the molecules for which the values of the coherence length are given, which is not the universal number of course.

We thank the reviewer for this remark and we add the name of the molecules (JC-1) when we refer to the values of the estimated N_{coh}. Considering this, the final sentence of the abstract will be:

"This approach revealed that N_{coh} of JC-1 J-aggregates ranges from 3 to 6".

4. Experimental details and measurement of the PL quantum yield should be given in SI.

Considering this judicious comment from the reviewer and we decided to add the whole new section to the SI (page S4):

"Quantum Yield estimation:

JC1 monomer quantum yield was estimated by photon emission comparison with Rhodamine 6G (Φ=0.95). To obtain comparable solutions, the absorbance spectrum of both dyes must have the same intensity at the excitation wavelength (laser 485 nm). This is shown in Figure S4.

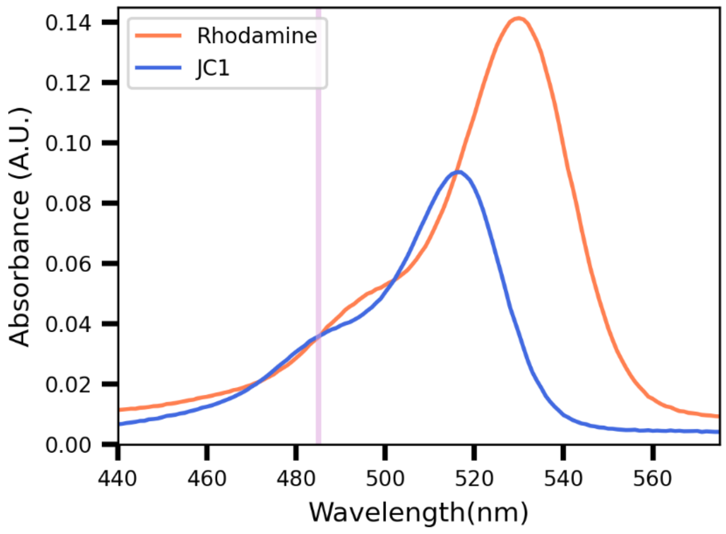

Figure S4: JC1 (blue) and Rhodamine 6G (orange) absorbance spectra in ethanol. The light purple line shows the used laser in PL measurements for sample's excitation at 485 nm.

Then, PL decay measurement of both solutions was performed. The area under the curve represents the emitted photon, we proceeded to integrate both spectra to quantify them.

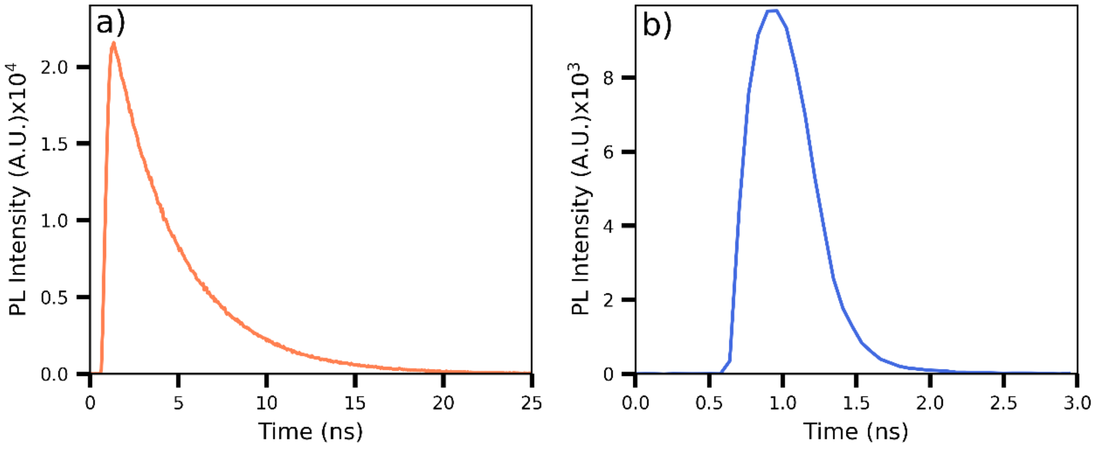

Figure S5: Photoluminescence decay measurement of a) Rhodamine 6G (orange) and b) JC1 monomer (blue) for photon emission count.

The Quantum Yield is defined as the relation between the absorbed and emitted photons. Since both PL measurements were performed under the same conditions and absorbance is equal at 485 nm, we can assure the absorbed photons quantity is the same in both cases and it is possible to relate ΦRh6G=0.95 and ΦJC1 and estimate the value for ΦJC1 = 0.05.

To calculate J-aggregates' QY we used Rhodamine 6G and a solution of core (CdSe)-shell (ZnS/CdS/ZnS) quantum dots. We applied the following equation to find the QY value using a reference sample (1):

$$\Phi_{f,x} = \Phi_{f,st} \frac{F_x}{F_{st}} \frac{f_{st}(\lambda_{ex})}{f_x(\lambda_{ex})} \frac{n_x^2}{n_{st}^2} \qquad (S1)$$

Where F is the spectrally integrated photon flux given by the integrated area under the emission spectra, f represents the absorption factor and n the refractive index of the solvent (1). The 'st' sub index refers to the standard solution with known QY.

To estimate F, we used the following equation (S1):

$$F = \frac{1}{hc_0}\int_{\lambda_1}^{\lambda_2} I_c(\lambda_{ex}, \lambda_{em})\lambda_{em}\, d\lambda_{em} \qquad (S2)$$

The fraction of the excitation light absorbed by the sample was estimated using:

$$f(\lambda_{ex}) = 1 - 10^{-A(\lambda_{ex})} \qquad (S3)$$

Where A represents the absorbance value at the excitation wavelength.

The Rhodamine 6G was in ethanol solution (n = 1.36), QDs were in hexane solution (n = 1.37) and J-aggregates in water (n = 1.33).

The absorbance spectra of the solutions (Figure S6) were used to determine the parameter $A(\lambda_{exc})$, in the case of QDs' QY calculation the excitation wavelength was 490 nm and 580 nm for J-aggregates' QY.

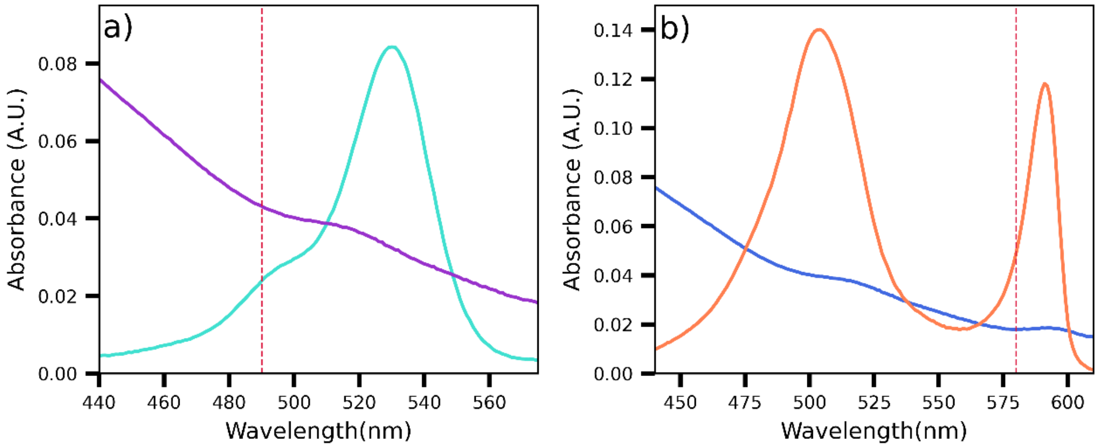

Figure S6: Absorption spectrum of Rhodamine 6G, QDs and J-aggregates. Part a) displays the Rhodamine 6G (light blue) and QDs (violet) absorbance response and the red dotted line shows the excitation wavelength at 490 nm. b) Absorbance spectra of J-aggregates and Quantum Dots. Dashed vertical line indicates excitation wavelength at 580 nm.

Once we established the excitation wavelength we measured the PL spectra to estimate parameter F (equation S2) and then, to apply equation (1). First, we calculated ΦQDs=0.4, and then ΦJ-agg=0.3.

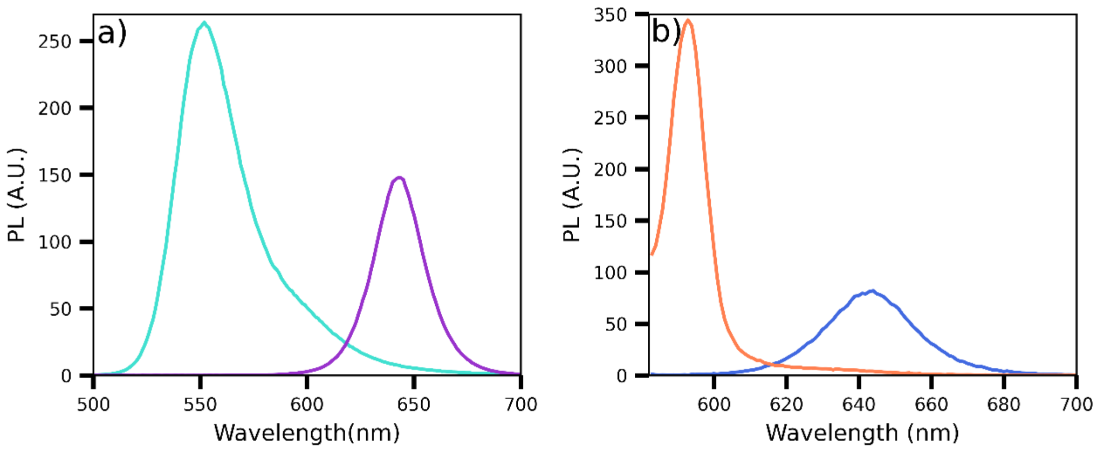

Figure S7: Photoluminescence spectra of Rhodamine 6G, QDs and J-aggregates. a) Rhodamine 6G (light blue) and QDs (violet) PL response with the excitation wavelength at 490 nm. b) Photoluminescence spectra (excitation at 580 nm) of J-aggregates (orange) and Quantum Dots (blue).

3. In conclusion, after the major revision the manuscript could be appropriate for more specialized journal.

We are grateful to the reviewer for careful and detailed comments on our work and for the overall favorable assessment. We would have considered a more specialized journal, but our findings encompass data from many different experimental techniques and phenomena. We would like to bring this work to as broader as possible audience of researchers and believe that the Journal of Physical Chemistry Letters is the most suitable platform for this.

Reviewer #2

1. The manuscript reports on various methods to determine the coherence length in isocyanine J-aggregates, highlighting the various methods used and the variation in the coherence numbers obtained. It is clearly written and the subject is very timely, since the coherence length of excitons plays a very important role in energy transport for applications such as solar cells. Coherence is also a central topic in quantum computing. The paper should be published subject to a successful response to the following:
1) In eq.(2) is the coupling J an effective nearest-neighbor coupling?

We are grateful to the reviewer for providing us his careful and detailed comments on our work and for the overall favorable assessment. To answer to this comment, it is necessary to define the parameter J, it is the dipole-dipole interaction strength in a molecular chain. In our case, we consider two molecules n, m in the J aggregates chain, where the coupling interaction will be described by the transition dipoles (μn, μm) as follows:

$$J_{m,n} = \frac{1}{4\pi\epsilon_0}\left(\frac{\mu_m\,\mu_n}{\left|r_{m,n}\right|^3} - \frac{(\mu_m r_{m,n})(\,\mu_n r_{m,n})}{\left|r_{m,n}\right|^5}\right)$$

If we also consider the starting and end molecules, in the N-molecular chain, feel the same symmetric interaction to two neighbors we can generalize: J0, N-1 = JN-1,0 = Jm, n = J.

Then, we can say J is derived from the collective response of the molecules and it can be referred as an effective value if we define this term as the representative value of the collective molecular response.

2. Eq.(4) is approximate in that it assumes the HR factor is sufficiently large that the absorption and emission spectra can be modelled with Gaussian line shapes (with unresolved vibronic peaks). This may be worth a mention since the monomer HR factor from Fig 1a is quite small - maybe 0.3.

   In fact, the monomer HR factor is small, it has a value of 0.1. To determine it without considering line broadening and thermal occupation we used equation (3), to determine the term ΔE_{bc}. Then, we can estimate the barycenter energy difference between PL and absorbance response directly from the acquired spectra of the sample using equation (4).

3. There is a more recent method based on HR factors for determining the coherence number. Based on the monomer HR factor S_{mon} (for the vinyl stretching mode -around 0.18 eV) which can readily be determined from the spectra in Fig.1a, the coherence number is given by $N_{coh} = \{I0\text{-}0 / I0\text{-}1\} * S_{mon}$ where I0-0 is the line strength of the 0-0 transition in the J-aggregate PL spectrum and I0-1 is line strength of the first side-band in the PL spectrum. (I0-0 and I0-1 refer to the peak intensities in a spectrum where the cubic dependence on the photon frequency has been deconvoluted away). Details appear in,
   1) Spano, FC and Yamagata, H May 12 2011 | JOURNAL OF PHYSICAL CHEMISTRY B 115 (18), pp.5133-5143
   2) Hestand, NJ and Spano, FC Aug 8 2018 | CHEMICAL REVIEWS 118 (15), pp.7069-7163

   The authors should at least mention this method.

   We appreciate this comment about this relevant PL related N_{coh} estimation method and the provided references. The presented method relates N_{coh} with temperature and disorder (σ). It is stablished that it is possible to apply the line strength ratio to evaluate exciton coherence length as long as kb.T, σ<< ω (34). Although this method presents a very reliable way to obtain the exciton coherence length, the study of coherence length on temperature and extent of disorder is beyond the scope of our work and these parameters are not among the variables we consider. We plan to do such research in the future. To reflect the commented method, we have added to the manuscript the following text in page 14:

   ”There is another method related to PL response and N_{coh}. It is stablished that it is possible to apply the PL line strength ratio to evaluate exciton coherence length at room temperature as long as kb.T, σ(disorder)<< ω (34). Although this method presents a very reliable way to obtain the exciton coherence length, the study of coherence length on temperature and extent of disorder is beyond the scope of our work and these parameters are not among the variables we consider. We plan to do such research in the future.”

jz-2022-022136.R2

Name: Peer Review Information for "Cross Determination of Exciton Coherence Length in J-aggregates"

Second Round of Reviewer Comments

Reviewer: 3

Comments to the Author

This manuscript reports the estimation of the excitonic coherence length for J-aggregates of a cyanine dye. Different spectroscopic methods have been compared, including absorption, fluorescence, time-resolved photoluminescence, and transient absorption. The authors concluded that a combination of these methods is required to obtain a reliable result. As different methods have been used in literature, such a comparison work is worth publishing. I recommend this work be published after addressing the following two points:

1) As many methods are compared in the manuscript, a table that lists the assumptions, the pros and cons of different methods should be provided to better guide the readers.

2) One question remained to be answered in the manuscript is that why the coherence length of the cyanine aggregates changes with pH.

Author's Response to Peer Review Comments:

Prof. Yury Rakovich
Ikerbasque Research Professor
Materials Physics Center
Paseo Manuel Lardizábal 5
Donostia-San Sebastián, Spain

October 18, 2022

Manuscript ID: jz-2022-022136

Prof. Editor
Senior Editor
The Journal of Physical Chemistry Letters

Dear Dr. Editor,

We would like to thank the reviewer for helping us to improve our manuscript with his/her comments. We found the assessment to be positive and valuable. Taking all the reviewer's comments into account, we reinforced our manuscript and developed a revised version. We hope that our improvements and explanations will be found complete and satisfactory.

Sincerely yours

Yury Rakovich

Replies to reviewer #3:

Comments:

This manuscript reports the estimation of the excitonic coherence length for J-aggregates of a cyanine dye. Different spectroscopic methods have been compared, including absorption, fluorescence, time-resolved photoluminescence, and transient absorption. The authors concluded that a combination of these methods is required to obtain a reliable result. As different methods have been used in literature, such a comparison work is worth publishing. I recommend this work be published after addressing the following two points:

1. As many methods are compared in the manuscript, a table that lists the assumptions, the pros and cons of different methods should be provided to better guide the readers.

   We thank the reviewer for his remark, we decided to add a table at the end of the results discussion section to summarize the obtained values and used techniques.

   We added the following table on page 6:

Table 1: Table summarizing the applied methods for JC-1 J-aggregates N_{coh} determination: N_{coh} value, advantages, shortcomings and references.

**Technique**	**N_{coh}**	**Advantages**	**Shortcomings**	**Ref.**
Absorbance J-band's linewidth	10.5	Only requires absorbance spectrum.	Error from J-band linewidth estimation.	9
Huang-Rhys parameter	3	Avoid thermal effects influence.	Considers absorbance and PL line shapes mirror-image symmetric.	14, 27
PL lifetimes	3	Only PL properties analyzed: lifetimes, quantum yield and emission wavelengths.	Warping effects on the molecules.	12, 28-30
Transient absorption	5.3	Only requires TA spectrum.	Spectrum saturation and sophisticated equipment is required.	16, 35

2. One question remained to be answered in the manuscript is that why the coherence length of the cyanine aggregates changes with pH.

We appreciate this comment pointing out the pH relation with the exciton coherence length value, to justify this relation we added the following text in page 16:

"The pH relation with the exciton coherence length of J-aggregates can be explained by their oxidation. The J-aggregates become oxidized in acidic environments (small pH values), which leads to a drop in absorbance and fluorescence. This makes acidic conditions inherently disruptive for the coherence length. In contrast, oxidation becomes no longer possible under basic conditions (up to about pH 10), where we found that the N_{coh} value stabilises."
